# A new use of Agrobacterium plant growth regulator genes for plant bioengineering

**DOI:** 10.3389/fpls.2026.1754357

**Published:** 2026-03-16

**Authors:** Michelle Heck, Marco Pitino, Samuel Coradetti, Stacy L. DeBlasio, W. Rodney Cooper, Lauren Shrum, Douglas Harper, Martin Stallone, Aspen Scott, Rachel Cook, Brian Rhodes, Samantha Sullivan, Elijah Schechter, Ellen Cochrane, Nicholas Larson, Guilherme Locatelli, Joanne Hodge, Magali Ferrari Grando, Li Wang, Meneka Ariyarante, Redeat Tibebu, Richard Stange, Kevin J. Howe, Ariana Makar, Douglas Stuehler, Luke Thompson, Ketan Shende, Matthew Hentz, Nichole Gaza, Chase Weeks-Purdy, Brian Chang, Ali Nikoomanzar, Lucy Bennett, Nursena Demirden, Wayne Hunter, James Thomson, Mark A. Ritenour, Lorenzo Rossi, Liliana M. Cano, Robert C. Adair, Eddie Stover, Cindy L. McKenzie, Randall Niedz, Robert G. Shatters

**Affiliations:** 1 United States Department of Agriculture (USDA) Agricultural Research Service, Emerging Pests and Pathogens Research Unit, Ithaca, NY, United States; 2 Plant Pathology and Plant Microbe Biology Section, Cornell University School of Integrative Plant Science, Ithaca, NY, United States; 3 Plant Pathology Department, Indian River Research and Education Center, Institute of Food and Agricultural Life Sciences, University of Florida, Fort Pierce, FL, United States; 4 United States Department of Agriculture (USDA) Agricultural Research Service, Temperate Tree Fruit and Vegetable Research, Wapato, WA, United States; 5 Indian River State College, Fort Pierce, FL, United States; 6 Tompkins Seneca and Cayuga BOCES New Visions Program, Ithaca, NY, United States; 7 United States Department of Agriculture (USDA) Agricultural Research Service Horticultural Research Lab, Fort Pierce, FL, United States; 8 Oak Ridge Institute for Science and Education, United States Department of Agriculture (USDA) Emerging Pests and Pathogens Research Unit, Ithaca, NY, United States; 9 Horticultural Sciences Department, Indian River Research and Education Center, Institute of Food and Agricultural Sciences, University of Florida, Fort Pierce, FL, United States; 10 Oak Ridge Institute for Science and Education, United States Department of Agriculture (USDA) Horticultural Research Lab, Fort Pierce, FL, United States; 11 United States Department of Agriculture (USDA) Agricultural Research Service, Crop Improvement and Genetics Research Unit, Albany, CA, United States; 12 Telesis Bio, San Diego, CA, United States; 13 Florida Research Center for Agricultural Sustainability, Vero Beach, FL, United States

**Keywords:** *Agrobacterium tumefacians*, biofactory, citrus greening disease (Huanglongbing), gall, genetic transformation, plant biotechnology

## Abstract

Delivery of biomolecules into plant vascular tissues remains a barrier to managing diseases caused by insect vector-borne pathogens and to modifying phenotypes of established perennial crops. Inspired by the vascularized growth of crown galls induced by *Agrobacterium tumefaciens*, we repurposed the bacterium’s plant growth regulator (PGR) genes to engineer autonomously dividing, transgene-expressing plant cell structures termed symbionts. A plant transformation vector (pSYM) incorporating the IaaM, IaaH, Ipt and gene5 cassette from *A. tumefaciens* strain C58 together with a gene of interest on the same transfer DNA was delivered to stems of herbaceous and woody dicots using disarmed *A. tumefaciens* strain EHA105. Symbiont morphology, vascular differentiation, transgene expression, molecular mobility and protein secretion were evaluated using microscopy, fluorescent reporters, dye tracing, RNA silencing assays and mass spectrometry-based proteomics. pSym inoculation reproducibly generated symbionts across diverse host plant species that were vascularly integrated into their host plants and transgene expression ranging from heterogeneous niches to more uniform patterns. Small molecules moved between symbionts and host vascular tissues, whereas larger proteins exhibited more restricted mobility. Post-transcriptional gene silencing signals moved freely throughout the symbiont and slightly into adjacent stem tissue. Under tested field and greenhouse conditions in potato and tomato, respectively, gall or symbiont formation had no negative impacts on plant growth or tuber and fruit yield. *In vitro*, symbiont cultures abundantly secreted recombinant protein into surrounding media. Together, these results establish symbionts as a modular, plant bioengineering platform capable of producing and potentially delivering biomolecules without modifying the host plant genome, providing a foundation for vascular-targeted therapeutics and phenotype modulation in crops.

## Highlight

Inspired by the challenge of solving citrus greening disease, we conceptualized and advanced research and development on a platform biotechnology with the goal of expressing and delivering antimicrobial peptides and other biomolecules to citrus vascular tissues by leveraging the natural process of gall formation that occurs during the development of crown gall disease caused by the plant pathogenic bacterium *Agrobacterium tumefaciens*. Using a plant transformation vector to express *Agrobacterium* plant growth regulator genes together with a gene of interest in the same cells, we engineered autonomously dividing plant cells, referred to as symbionts (to distinguish from pathogenic galls), capable of expressing biomolecules on plants and *in vitro* plant cell tissue culture. This new application of plant growth regulator genes from *A. tumefaciens* can potentially be used to modify host plant phenotype, including physiological, morphological traits, biochemical or other plant characteristics, without modification of the host genotype with future research.

## Introduction

**Figure 1:**
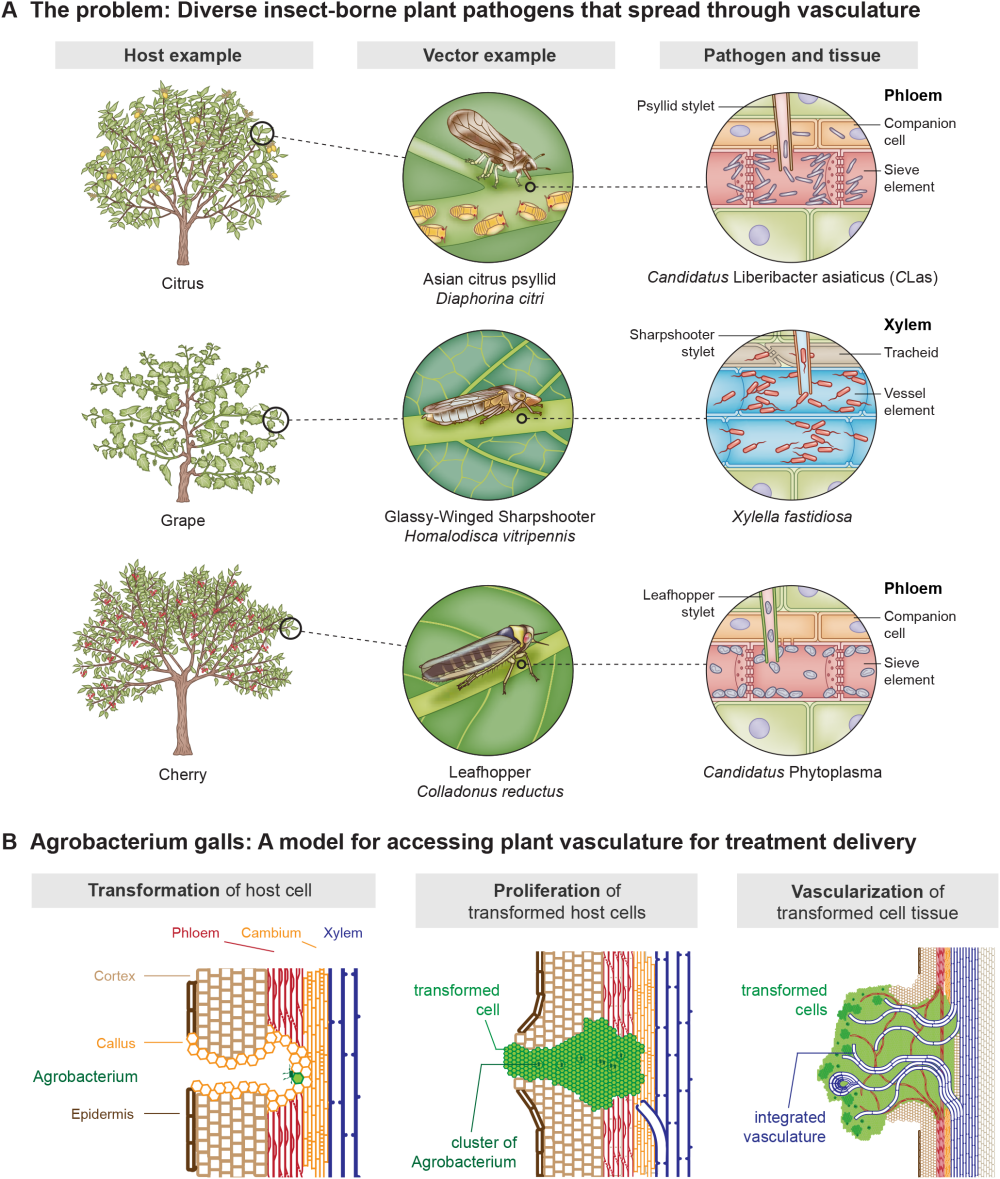
Diverse insect vector-borne plant pathogens spread through the plant through the vascular tissue. **(A)***Candidatus* Liberibacter asiaticus is a bacterium associated with citrus greening disease, a devastating condition in citrus trees. This pathogen is spread by at least two psyllid species, including the Asian citrus psyllid (*Diaphorina citri*), which transmits the bacteria as it feeds on phloem tissue, leading to fruit drop, stunted growth, and eventual tree death. Similarly, *Xylella fastidiosa*, the causative agent of Pierce’s disease in grapevines, is transmitted by the glassy-winged sharpshooter (*Homalodisca vitripennis*). This xylem-restricted bacterium clogs the water transport systems in vines, causing wilting, dieback, and significant economic losses in viticulture. *Colladonas reductus* is a leafhopper known to transmit phytoplasmas, which cause diseases like Cherry X disease in cherries. These phytoplasmas disrupt phloem function, resulting in yellowing, leaf curl, and reduced fruit production. Collectively, these vector-pathogen interactions in the plant vascular tissue highlight the critical threat posed by these plant pathogens to global agriculture and the need for technologies that target therapies to the plant vascular tissue. **(B)***Agrobacterium tumefaciens* is a soil-dwelling bacterium and the causative agent of crown gall disease, characterized by tumor-like growths at the crown or lower stems of a wide range of plants. The bacterium initiates gall formation by transferring a segment of its Ti plasmid DNA (T-DNA) into the host plant cells, where it integrates into the plant genome. This T-DNA encodes genes that stimulate uncontrolled cell proliferation and the production of opines, which the bacterium uses as a unique carbon and nitrogen source. The developing gall recruits vascular tissue from the host plant, forming a network that facilitates nutrient and water transport into the growing structure. This vascular integration supports the gall’s metabolic demands, effectively turning the host plant into a nutrient source for the tumor and the bacterium, highlighting *A tumefaciens*’ unique evolutionary adaptation as a plant pathogen. The biology of the gall was the inspiration for the development of the symbiont concept.

Insect vector-borne bacterial pathogens are responsible for significant crop losses globally. Key examples include genera such as *Xylella*, *Candidatus* Liberibacter asiaticus (*C*Las), *Spiroplasma*, and *Candidatus* Phytoplasma. *Spiroplasma*, and *Ca. Phytoplasma* species are restricted to the phloem, where they colonize sieve elements (**Figure 1A**). In contrast, *X. fastidiosa* colonizes the xylem tissue (**Figure 1A**). These bacteria are transmitted by diverse piercing-sucking insects within the order Hemiptera (**Figure 1A** (Huang et al., 2020). Farmers producing citrus, grapes, cherries, almonds, potatoes, and olives have faced serious challenges managing these pathogens in recent years.

Targeting therapies directly to the vascular tissue has the highest potential to provide symptom relief to vascular pathogens (Li et al., 2019). Florida growers are now using injectable oxytetracycline (OTC) to individually treat trees infected with *C*Las to manage citrus greening disease (Albrecht and Batuman, 2024; Chakravarty and Wade, 2024; Albrecht et al., 2025). Antimicrobial peptides (AMPs) sourced from plants and other organisms have been shown to disrupt the *C*Las life cycle (Huang et al., 2021; Higgins et al., 2024). Such AMPs would have a favorable and expedited regulatory path compared to medically-important antibiotics or new synthetic pesticides. AMPs could be used in combination with antibiotics to minimize the development of resistance and increase treatment efficacy (Hegreness et al., 2008; Lehár et al., 2009; Cassone and Otvos, 2010; Cokol et al., 2011; Shah and Chen, 2017; Greve and Cowan, 2022; Lennard et al., 2023). However, AMPs are currently impractical to source and deliver at the quantities required for grove-level, direct injection or foliar application. New technologies are needed for widespread use and adoption of AMPs and other biologically-based plant protection products in agriculture, in particular technologies that can deliver therapies directly into the tree’s vascular tissues.

To meet this need, our team developed a new use for the plant growth regulator genes from *Agrobacterium tumefaciens. A. tumefaciens* is the bacterium that induces crown gall disease and emerged as the foundation of plant genetic engineering in the late 1970s. Wild isolates of the *Agrobacterium* genus harbor a diverse range of transfer DNAs (T-DNAs), segments from the bacterium’s tumor-inducing (Ti) plasmid that are transferred into the plant nuclear genome. T-DNAs encode enzymes to condense amino acids and sugars or ketones into opines, the major source of carbon and nitrogen for the infecting bacteria (Petit et al., 1983). The range of different enzymes found on T-DNAs (Vladimirov et al., 2015) and opines (Moore et al., 1997) associated with *Agrobacterium* infection on various plants is wide. To facilitate the production of opines, T-DNAs also encode plant growth regulatory genes (PGR genes), which stimulate the formation of a gall, a tumor-like assemblage of autonomously growing cells at the infection site (**Figure 1B**). The best studied PGR genes are those of the *A. tumefaciens* C58 Ti plasmid, consisting of three core genes: *IaaH*, *IaaM*, and *Ipt* (Akiyoshi et al., 1983; Joos et al., 1983; Zhang et al., 2015). Upon integration into the plant genome, *IaaH* and *IaaM* expression increases the local concentration of the plant growth hormone auxin (Teale et al., 2006). As auxin levels rise, auxin-responsive plant transcription factors, as well as pathogen-response transcription factors activate *Ipt* expression, which catalyzes the rate-limiting step in the synthesis of cytokinin (Werner et al., 2001). In addition to cytokinin synthesis, elevated auxin levels induce expression of an indole-3-lactate synthase (often named *gene5* in previous studies) on the T-DNA (Körber et al., 1991). Indole-3-lactate has been described as both an auxin analog (Sprunck et al., 1995), and an auxin antagonist (Körber et al., 1991).

Developing galls are supplied with nutrients and water through the recruitment of phloem and xylem, the vascular tissues, into the structure, a process that can be regulated by the PGR genes. Aloni and colleagues (Aloni et al., 1995) examined the vascular patterns in crown galls induced by *A. tumefaciens* in the stems of *Ricinus communis* (castor bean). They observed two types of vascular strands: branched, tree-like bundles in fast-growing regions and round bundles in slower-growing areas. Both types were observed to connect with the plant’s vascular system in the stem. They observed that phloem anastomoses, formed by sieve tubes, created a dense network linking the tumor phloem bundles. In the 1940s, Braun showed that plant cells removed from *A. tumefaciens* galls could be cultured *in-vitro* and cured of the bacterium (Braun, 1943). Critically, the cured cultures retained the capacity to form a vascularized gall when transplanted back to host plants. This finding demonstrated that while plant-microbe interactions likely influence some aspects of gall growth and morphology, the essential elements reprogramming plant development and vascular differentiation are wholly embodied in the genes integrated into the gall genome.

Here, we demonstrate how PGR genes can be used for biotechnological applications through the construction of a non-pathogenic gall of autonomously dividing cells that is distinct from, yet vascularly integrated with a host plant (**Figure 1B**). In keeping with generally accepted terminology for an autonomously dividing, genetically distinct entity that is dependent on a host for survival and also may provide an adaptive advantage to that host, we call this engineered organ a “symbiont”. Specifically, a symbiont is a transgenic plant cell (or cluster of cells) that harbors an integrated T-DNA expressing both PGR genes and a gene of interest within the same cell (He et al., 2020). Our currently pending patent application further defines the concept more technically, stating that a “symbiont” is a plant cell or plurality of plant cells comprising a polynucleotide encoding a phytohormone biosynthetic enzyme (e.g., at least one polynucleotide encoding one or more phytohormone biosynthetic enzymes and a polynucleotide of interest (Shatters et al., 2021). With future research, some of which is outlined in **Table 1** and described in the results section of our manuscript, we envision that these symbiotic cell clusters will be a flexible, modular platform to deliver beneficial proteins, antimicrobial peptides and small molecules to the host plant.

**Table 1:** Key areas of future research to optimize symbionts as crop protection tools.

Research area	Focus	Summary of challenge
1	Symbiont morphology is host-plant specific.	Experiments conducted on a range of plant species demonstrates variance in symbiont morphology within and among species. It is unclear whether a particular morphology is required to impart symbiont-induced changes to a host plant phenotype.
2	Symbionts were not formed on plants following biolistic bombardment of symbiont plasmids using a gene gun.	An interesting new frontier of research includes making symbionts on plants using methods that do not require the use of live *Agrobacterium* cells. Experiments to initiate symbiont formation on multiple plant species using a Helios gene gun to deliver symbiont plasmid DNA only (no bacteria) were unsuccessful. Future research is needed to understand the factors that led to failure and whether they can be overcome, for example by co-expressing *Agrobacterium* vir genes or suppressing plant cell death following bombardment injury.
3	Symbiont formation using transplantation was successful in tomato but not citrus despite multiple attempts.	Transplantation of *Agrobacterium*-free symbiont tissue from tissue culture plants to citrus trees failed to form symbionts over numerous experiments, while the same experiment worked successfully in tomato. Understanding the causes of failure in citrus will be important to further develop transplantation as a scalable commercial delivery system.
4	Transgene expression in symbionts is variable.	In all experiments, symbionts exhibited variation in the number of cells expressing the transgene on the symbiont plasmid. The reasons for the variation are yet unknown and might include variability in transformation efficiency, uneven cell proliferation within the structure, stem cell-type proliferation responses to hormone gradient and other possibilities.
5	Larger molecules are movement restricted within the symbiont and between the symbiont and the host plant vascular tissue.	Experiments using fluorescent proteins, dyes and siRNAs demonstrated that molecular size plays a critical role in determining whether the product will move freely throughout the symbiont structure and between the symbiont and host plant. Research to control symbiont export and control of molecule movement will be essential to deliver larger therapies, such as proteins, from symbionts to trees and limit symbiont-delivered therapies to smaller molecules, such as siRNAs and antimicrobial peptides.
6	Commercial scale delivery of the technology must be developed and scaled at cost.	Generating symbionts involves manual inoculation by lab personnel and is not ideally suited to row crops. Various aspects of delivering technology at scale and cost must be further developed for grower adoption.

## Materials and methods

### Construction of the pSym plasmid and derivative vectors used in this study

**Figure 2:**
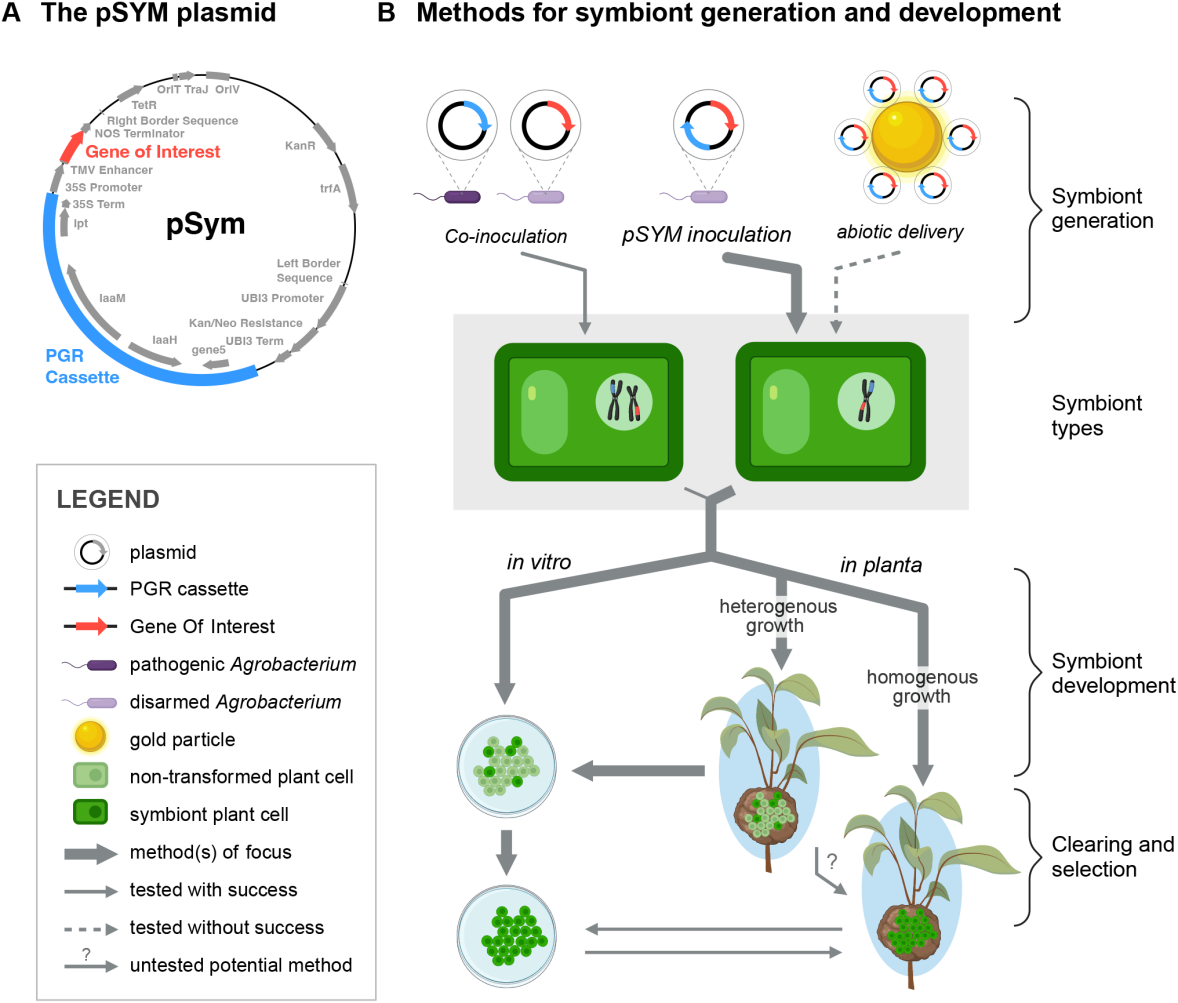
The molecular biology and general methodologies of symbiont formation. **(A)** The pSym plasmid encodes the PGR cassette genes ipt, IaaM, IaaH and gene 5 under their native promoters from *Agrobacterium tumefaciens* strain C58 together with a gene of interest under the control of an enhanced double 35S promoter. Kanamycin resistance genes are encoded both within the T-DNA (plant) and the vector backbone (bacteria). **(B)** There are various methods to generate a symbiont, and the transformation efficiency at the site of inoculation needed to form symbionts is unknown. Co-inoculation of a pathogenic *A tumefaciens* strain with a disarmed strain of *A tumefaciens* carrying a binary plant transformation vector can be used to generate symbionts comprised of cells harboring both a wild-type T-DNA with the PGR genes and a T-DNA encoding a gene of interest from the disarmed binary vector. Inoculation of the pSym plasmid carried by a disarmed strain of *A tumefaciens*, such as EHA105, reliably produces symbionts with the PGR cassette and the gene of interest within the same T-DNA integration. Inoculation of pSym using disarmed strains of *A tumefaciens* has been the major focus of our research efforts to date. Particle bombardment and other non-biologically based methods can theoretically be used to deliver the pSym plasmid into the plant nucleus, thus eliminating the need to rely on *A tumefaciens* but further work needs to be done to develop these strategies. Symbionts can be formed on plants or in tissue culture. On plants, symbionts may be used to deliver biomolecules to the plant or possibly as a biofactory to produce biomolecules to be harvested and used for other applications. Symbionts can be generated *in vitro* by transforming callose tissue with pSym plasmids or excised from the plant and grown in tissue culture. On plants, symbionts exhibit transgene expression along a continuum from heterogeneous in distinct cell niches to more homogeneous and uniform.

The pSym plasmid (**Figure 2A**) was made by PCR-amplifying the PGR cassette from *A. tumefaciens* strain C58 and ligating into pUSHRL-26 (Krystel et al., 2021) at the AscI site immediately upstream of the double 35S promoter (**Figure 2A**). An annotated, sequenced-confirmed map of pSym is available on GenBank (PQ563460). We built several versions of pSym for expression of the fluorescent marker proteins GFP and mCherry with leader secretion signal peptides (SP), and/or mobile plant protein domains (e.g. flowering locus T-like protein 3 from *Citrus clementina* (FT3) (Soares et al., 2020) in an attempt to direct movement of the protein of interest throughout the plant. Plasmid pSYM-FT-mCherry was used for the *in vitro* growth experiments. Another variant involved pSym-CsGAOX-mCherry-3xHA, co-expressing the citrus homolog of GA2oxidase 8 and mCherry and was used in the dye loading experiments. The pSYM plasmid can be obtained from the global non-profit microbial resource center, ATCC, upon request (www.atcc.org).

A smaller symbiont-forming vector was constructed by inserting the PGR cassette from pSym into the pJL-TRBO backbone (Lindbo, 2007) and placing the small molecule reporter RUBY (He et al., 2020) under the 35S promoter (pMin2-Ruby). To silence YFP expressed in host plant cells, a hairpin RNA complementary to the YFP sequence was inserted into pMin2 under the 35S promoter and the mCherry sequence was added under the NOS promoter to make pMin2_hpYFP_mCherry. Annotated sequences for these vectors are available for download on GitHub (https://github.com/MichelleHeck77/symbiont-plasmids).

### Symbiont inoculation using *A. tumefaciens*

EHA105 cells carrying pSym plasmids were grown overnight in LB containing kanamycin at 50-100 µg/ml at 28 °C at 200 rpm, reaching approximately 1.0 OD_600_. Cells were washed two to three times in an infiltration buffer (10 mM MES, 10 mM MgCl_2_ 400 µM acetosyringone, pH 5.6) then incubated in this buffer for at least 1 hour at room temperature to activate the *A. tumefaciens*. To inoculate herbaceous plants such as *Arabidopsis*, potato, or tomato, an insulin syringe was used to deliver droplets of the symbiont forming inoculum into the stem at 1.0 OD_600_. For citrus and all other woody plants, a small piece of bark was excised using a biopsy punch (1–6 mm) to expose the vascular cambium and other underlying cells and create a consistent inoculation site. Approximately 20 µl of the Agrobacterium inoculum was then delivered to this site, also at 1.0 OD_600_. The inoculation sites were wrapped in parafilm to maintain moisture, then a secondary wrap such as black K-Flex insulation foam, black silicon tape, or vet wrap, to protect the inoculation site from light.

### Vascular tissue imaging

Generally following the protocol outlined in (Parker et al., 1982), at 7 or 12 weeks post inoculation, 1–2 millimeter longitudinal sections of unwrapped, pSym- and wild-type *A. tumefaciens* (strain ID159)-induced stem galls on potato (*S. tuberosum* ‘Desiree’) were obtained manually using a vegetable mandolin slicer and quickly rinsed in ddH_2_O. Sections were incubated for 2–3 minutes in a 1:3 dilution of a 0.05% toluidine blue stock solution solubilized in ddH_2_O. Stained sections were washed 5-6x by incubating sections for 1–2 min in 5 mL of ddH_2_O and successively transferring sections with tweezers to fresh aliquots of ddH_2_O. Each section was immediately imaged under dark field conditions on a Leica M205-A stereo microscope using multi-focus, Z-stack parameters with white-balance. Brightness, contrast, tone and saturation of each image were enhanced using Photoshop on all images uniformly.

### Growth of symbiont tissue *in vitro*

Tomato plants (cultivar ‘Lanai’) were inoculated with the symbiont-forming *A. tumefaciens* strain EHA105 carrying pSym plasmids as described above. One to two months post-inoculation, symbionts were excised from the plant stem using a sterile scalpel. Excised symbionts were surface-sterilized by sequential immersion in 70% ethanol for one minute, followed by 10% bleach (0.6% sodium hypochlorite) for 10 minutes and rotated in the solution during this time period. The symbionts were then rinsed thoroughly with sterile distilled water three times to remove residual sterilizing agents. Sterilized symbionts were placed on solid culture medium consisting of Murashige and Skoog (MS) basal salts with vitamins (4.33 g/L), sucrose (37 g/L), and Gelrite (2 g/L), the pH of the medium was adjusted to 5.7. To eliminate any residual *A. tumefaciens*, the medium was supplemented with cefotaxime (100 mg/L), vancomycin (100 mg/L), and timentin (100 mg/L). Kanamycin (50 mg/L) was also included to maintain selection for the pSym plasmid within the symbionts. Symbiont cultures were incubated at 25°/27 °C in the dark.

### Mass spectrometry-based proteomics of symbiont tissue

Tomato symbiont tissue homogeneously expressing FT-mCherry fused to a secretion signal was transferred to fresh plates (same components as above) and the residual liquid media on these plates was sampled over time at different intervals, 3, 6 or 12 days, or for a total of 30 days to test the hypothesis that symbiont cells are actively secreting protein product into the medium on the plates and to determine whether there is an optimum collection frequency at which the product of interest would maximally accumulate in the liquid media. Symbiont tissue was sampled at time point zero on each plate. A total of 1 ml of the liquid media accumulated around the symbiont cells in the agar media was collected for each time point. Proteins were extracted using trichloroacetic acid (TCA) and acetone with 2% beta-mercaptoethanol as described in (Cilia et al., 2009). Symbiont tissue was cryogenically lysed using a MM 400 Mixer Mill (Retsch) before protein precipitation for a total of 3 min (3 x 1 min time interval), whereas the proteins from the liquid media were precipitated directly into the 10% TCA-acetone solution. Samples were prepared for mass spectrometry and analyzed as described in (DeBlasio et al., 2021) on a Q Exactive mass spectrometer (Thermo Fisher Scientific). A tomato protein database was downloaded from solgenomics.net and modified to contain the FT-mCherry sequence for protein identification in 2022. Raw data was converted into *.mgf files using msConvert from Proteowizard (Chambers et al., 2012) and searched against the database using Mascot Daemon (Perkins et al., 1999) with a precursor tolerance of 20 ppm, fragment tolerance of 0.6 Daltons. Methylthio was selected as a fixed modification on cysteine with 1 miss tryptic cleavage site. Deamidation of asparagine and glutamine and oxidation of methionine were selected as variable modifications. Total spectral counts were used to measure the relative abundances of each protein in the mixture over time. The mass spectrometry proteomics data have been deposited to the ProteomeXchange Consortium via the PRIDE (Perez-Riverol et al., 2025) partner repository with the dataset identifier PXD071454 and 10.6019/PXD071454.

### Carboxyfluorescein treatment of potato stems and symbionts

Vegetatively propagated potato plants (*Solanum tuberosum*, ‘Desiree’) were inoculated on the main stem ~3 weeks post rooting with *A. tumefaciens* EHA105 harboring pSym-CsGAOX-mCherry-3xHA. Inoculation was done by making three horizontal scores across the stem, approximately 0.5–1 mm apart, without going all the way through the stem. *A. tumefaciens* inoculum was then dripped over the wounds and the inoculation site immediately wrapped with parafilm. After a week of growth, the parafilm was removed. At 59 days post inoculation, a longitudinal cut site was made along the periphery of each symbiont, no more than 1–2 millimeters deep. Three square (~1x1 cm) pieces of Whatman filter (110 mm, Cat No. 1001 110) paper soaked in 0.65 mM 5 (Huang et al., 2021)-Carboxyfluorescien diacetate (CFDA, Sigma Aldrich Cat No. 21879) diluted into ddH2O from a 13 mM stock dissolved in 100% acetone, were immediately applied to cover the cut site. As a positive control, CFDA soaked filter paper was also applied to cut potato stems that were not inoculated with symbionts. Application sites were completely covered with parafilm and then wrapped with a piece of black Nashua Self-Fusing Silicone Stretch and Seal Tape. After 48 h incubation with CFDA, potato stems with or without symbionts were cut longitudinally down the middle across the CFDA application site and imaged live using a Leica M205 A epifluorescence stereoscope with the following filter sets: DAPI, LP GFP, and GFP. All images were taken with the same exposure and gain parameters for each filter set, which were set using negative control potato stems where CFDA was not applied. Final images were an overlay of each fluorescent channel. Several images were taken along a single stem and manually stitched together using landmark stem features. Images were post-processed in the LASX software for brightness and contrast, applying the same parameters to all images.

### Post transcriptional gene silencing in *A. thaliana* symbionts

We chose the pPIN4:4xYFP transgenic line of *A. thaliana* (Marquès-Bueno et al., 2016) to test efficacy of and extent of post-transcriptional gene silencing in symbionts because this line produces symbionts with relatively even YFP expression across the entire volume of the symbiont. Approximately four weeks post germination, seedlings were inoculated on the main inflorescence with *A. tumefaciens* EHA105 bearing pMin2_hpYFP_mCherry, expressing a hairpin RNA against the first 400 bp of the YFP sequence introduced by Marques-Bueno et al. and monomeric mCherry. After 4 weeks of symbiont growth in the greenhouse, a contiguous region of 1–2 cm of interconnected symbiont growth was obtained on each fluorescence. Symbionts were cut into thin cross sections and examined without fixation on a Leica M205 epifluorescence stereoscope. All images were subjected to identical excitation intensity, magnification, exposure and gain parameters. Symbiont vs stem regions of the cross sections were identified manually in FIJI (Schindelin et al., 2012). These regions were analyzed for fluorescence intensity in python 3.11.17 with python image library (ver 9.4.0), numpy (1.24.3), scipy (1.10.1), and seaborn (0.12.2) packages. As each cross section represented a sample of a contiguous (and thus not independent) region of symbiont growth, YFP intensity was averaged over all the analyzed sections for each plant and statistical comparisons were made between averages obtained from independent plants. To compare between multiple experiments on slightly differently aged plants with some differences in image acquisition, plant-average YFP intensities were normalized to the mean intensity observed in control plants for that experiment. The statistical test used was a two-way ANOVA. Differences between treatments had highly significant *P* values (less than 0.001), but between-experiments and interaction *P* values were all greater than 0.1.

## Results

### The symbiont concept is a platform technology for biomolecule production and delivery

Our initial proof of concept experiments started by mixing pathogenic strains of *A. tumefaciens* with disarmed strains, such as EHA105, harboring plant transformation binary vectors (**Figure 2A**). Inoculation of plant stems with these mixtures resulted in the generation of galls with clusters of cells expressing a transgene. To explore the potential of symbionts as a platform for rapid engineering of plants using standard laboratory, disarmed *A. tumefaciens* strains, we developed the vector pSym (**Figure 2A**). We generated pSym by integrating the PGR region from the Ti plasmid of *A. tumefaciens* strain C58 (including the coding sequences for *IaaM, IaaH, Ipt*, and *Gene5* under their native promoters) into the binary vector pUSHRL-26 (Krystel et al., 2021). The pSym plasmid (GenBank PQ563460) includes a cloning site for convenient transgene expression under an enhanced double CaMV 35s promoter and NOS terminator (**Figure 2A**).

**Figure 3:**
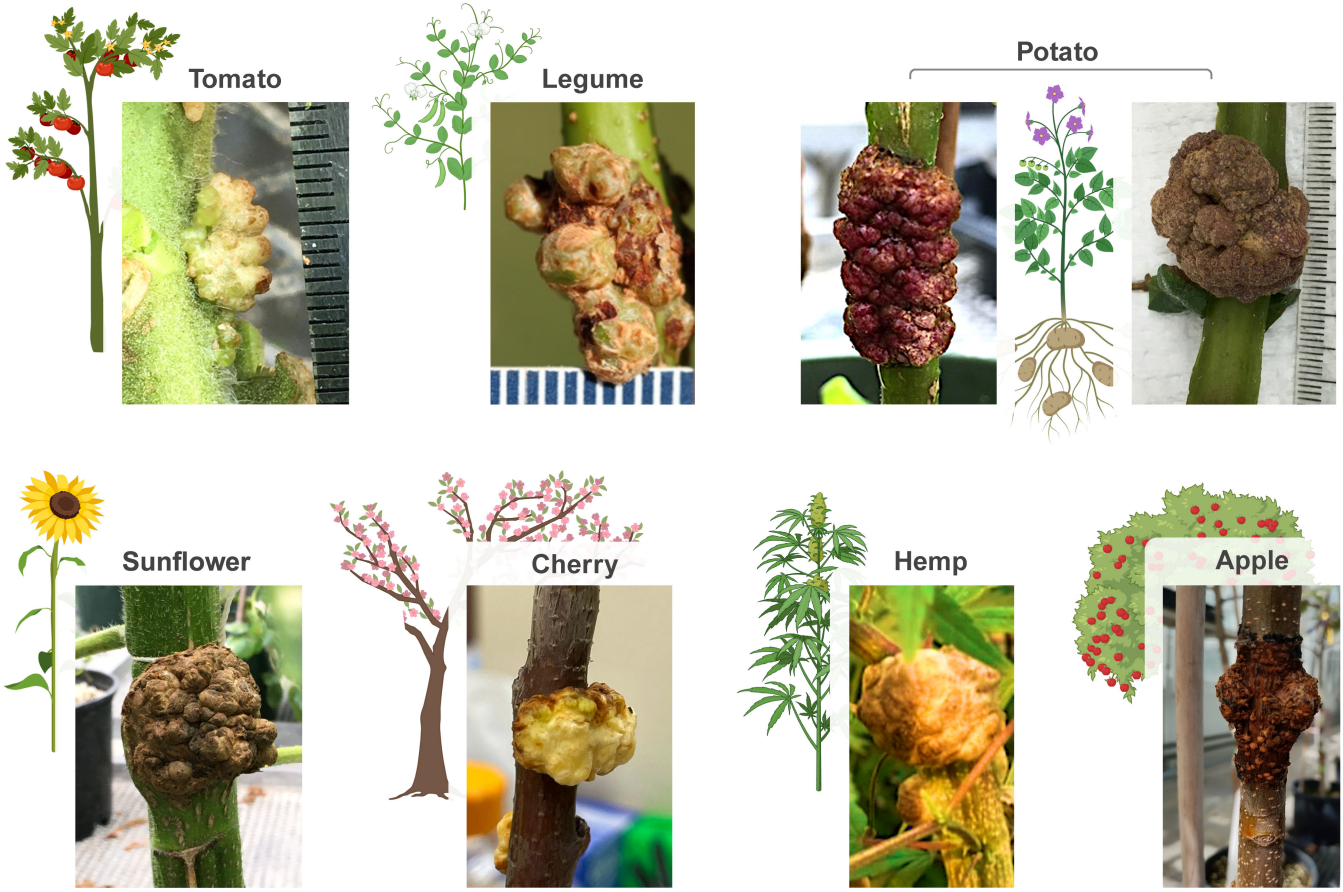
Expression of wild-type *Agrobacterium tumefaciens* plant growth regulatory genes (PGRs) in a binary plant transformation vector coupled to inoculation of the cambial tissue layer within stems using a disarmed strain of *A. tumefaciens* phenocopies various morphologies of gall development in a range of dicot plant species. These gall-like structures are referred to as symbionts to reflect a potential beneficial relationship between the structure and the host plant, which are genetically distinct due to the integration of the transgene in symbiont cells. Shown here are examples of symbionts formed on tomato, pea, potato, sunflower, cherry, hemp and apple using the pSym plasmid delivered by the disarmed strain of *A. tumefaciens*, EHA105.

Symbionts can be formed by inoculation of pSym-bearing *A. tumefaciens* directly on plant stems (**Figure 2B**). When various dicotyledonous plants were infiltrated with the disarmed *A. tumefaciens* strain EHA105 bearing pSym vectors, symbionts developed with broadly similar growth characteristics to galls (**Figure 3**). A full list of plant hosts that successfully supported symbiont formation from pSym include: apple, *Arabidopsis*, pea, citrus, cherry, cotton, grape, hemp, *Nicotiana benthamiana*, pecan, tomato, potato, sunflower, olive, almond, and blueberry. Furthermore, we observed different morphologies of pSym symbionts formed on these different plant species (**Figure 3**, **Table 1**). Methods for symbiont inoculation presented here were optimized for citrus, tomato, potato and *Arabidopsis*. For those species, over many dozens of experiments in five different laboratories, unambiguous symbiont growth was routinely observed for 60% - 90% of inoculations.

**Figure 4:**
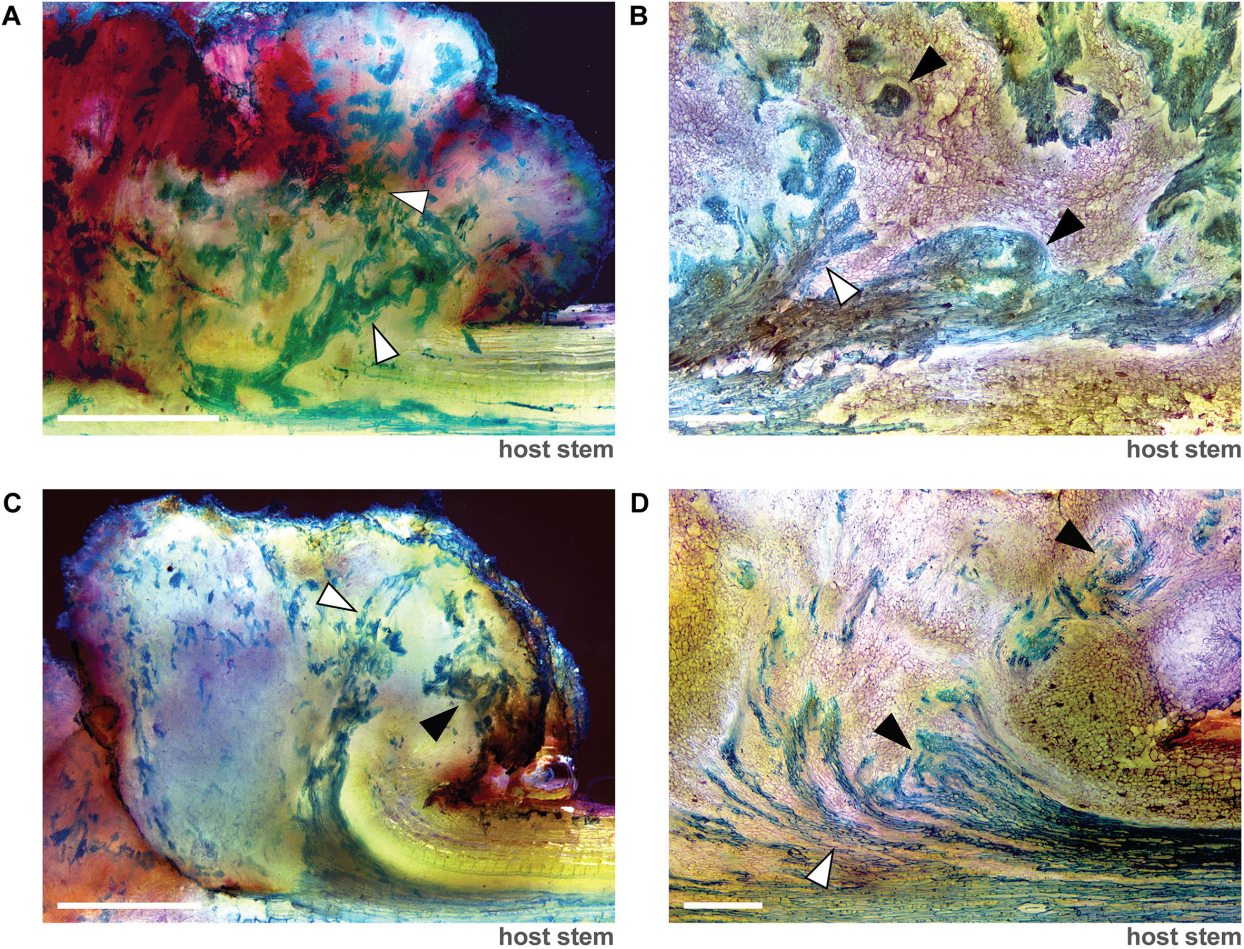
Vascular differentiation within symbiont structures is similar in patterning to wild-type *Agrobacterium*-induced gall growth. Representative, multi-focus Z-stacks of longitudinal sections taken from **(A, B)** wild-type *A. tumefaciens*-induced gall growth and **(C, D)** pSym-induced symbiont growth on potato (*S. tuberosum* ‘Desiree’) at **(A, C)** 7 weeks and **(B, D)** 12 weeks post infiltration stained with toluidine blue show both branched (white arrowheads) and globular (black arrowheads) xylem bundles (stained blue) connected to the main vascular bundle within the host stem. **(B, D)** Xylem bundles within the symbiont and galls are surrounded by parenchyma-like cells (stained purple). Red coloring indicates anthocyanin accumulation due to increased production of auxin within symbionts and potato galls. Images were obtained under dark field settings and scale bars = 500 µm.

Vascular differentiation within symbionts closely resembles the patterning observed in wild-type *A. tumefaciens*-induced galls (**Figure 4**). Longitudinal sections from both symbiont and wild-type galls on potato (*Solanum tuberosum*, c.v. Desirée) at 7- and 12-weeks post-inoculation reveal branched and globular xylem bundles connected to the main vascular bundle within the host stem. The xylem bundles are surrounded by parenchyma-like cells, and deep red pigmentation indicates anthocyanin accumulation linked to auxin production within the symbionts and galls (**Figure 4**). Symbionts developed on citrus or other wood plants must be wrapped in a vapor barrier such as parafilm for efficient symbiont formation (**Supplementary Figure S1**).

We performed several experiments using a Helios gene gun (Biorad) to deliver symbiont plasmids to stems (**Figure 2B**) and were unsuccessful in generating symbionts using this approach (data not shown, **Table 1**). Several factors may have prevented successful symbiont formation, including the high rate of cell death associated with particle bombardment, difficulty in targeting the specific cell layer most competent for symbiont formation, lack of *A. tumefaciens* vir gene expression, and a possible requirement for active cell division for stable DNA integration into the target genome. These challenges would need to be further investigated to determine whether use of bombardment may be a viable method to form symbionts.

### Viable *A. tumefaciens* is not necessary for symbiont formation after T-DNA transfer

In accordance with previous observations (Braun, 1943), the continued presence of *A. tumefaciens* was not a necessary condition for the maintenance of pSym-generated symbionts. To confirm this finding, we started by generating symbionts with pSym and the EHA105 *A. tumefaciens* strain on tomato stems, as described above. Then, we excised symbiont tissue from the plants onto plates with antibiotics to clear the agrobacterium. After several transfers on plates, we transplanted symbiont tissue back onto the plants. We were able to generate transplanted symbionts on tomato with some success (**Supplementary Figure S2**) as well as sunflower (not shown). However, in over 60 attempts across three independent experiments, we were unsuccessful in transplanting symbiont cells grown *in vitro* to initiate symbiont growth on citrus trees (**Table 1**). Symbiont growth using transplantation methods in tomato took significantly longer than direct inoculation using EHA105-mediated transformation of plant cells with the pSym plasmid, which is why we decided to focus further research efforts on developing symbionts using EHA105-mediated delivery of pSym plasmids (**Figure 2**).

### Transgene expression in symbiont tissue ranges from heterogeneous to homogeneous.

**Figure 5:**
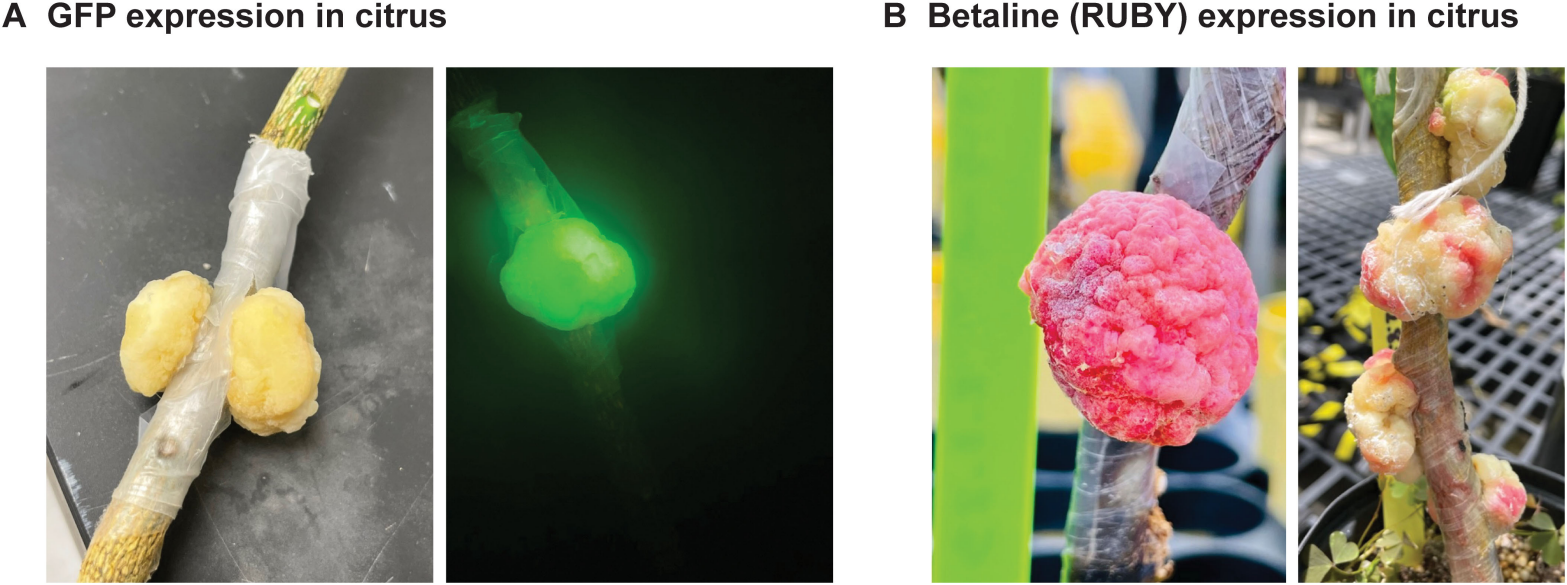
Expression of heterologous biomolecules in symbiont tissue on citrus. **(A)** Expression of GFP within a symbiont developing on citrus using brightfield (left) or fluorescence microscopy (right). **(B)** Expression of the RUBY reporter in symbionts developing on citrus. Shown is a symbiont homogeneously expressing RUBY (left) and symbionts that are heterogeneous for RUBY expression (right). Symbionts shown in both panels are approximately 4–6 weeks old.

Symbionts can be engineered to express proteins, such as GFP (**Figure 5A**). We used microscopy to explore fluorescent protein expression in symbionts. Symbionts were observed to contain several isolated niches of fluorophore-expressing cells at any one time (**Figures 5B** right, **6A**). From these data, it was not clear to what extent non-expressing cells within the growing symbiont were non-transformed host cells proliferating rapidly in response to local hormone gradients established by transformed cells; transformed host cells undergoing some form of gene silencing; or transformed cells that had integrated partial fragments of the pSym T-DNA not including the fluorophore gene. In some symbionts, transgene expression is more homogenous in the structure (**Figures 5B** left, **6B**; **Table 1**). Future work is required to understand the parameters that regulate transgene integration and heterologous gene expression in symbiont tissue. We hypothesize homogenous expression could be important for optimal symbiont function.

**Figure 6:**
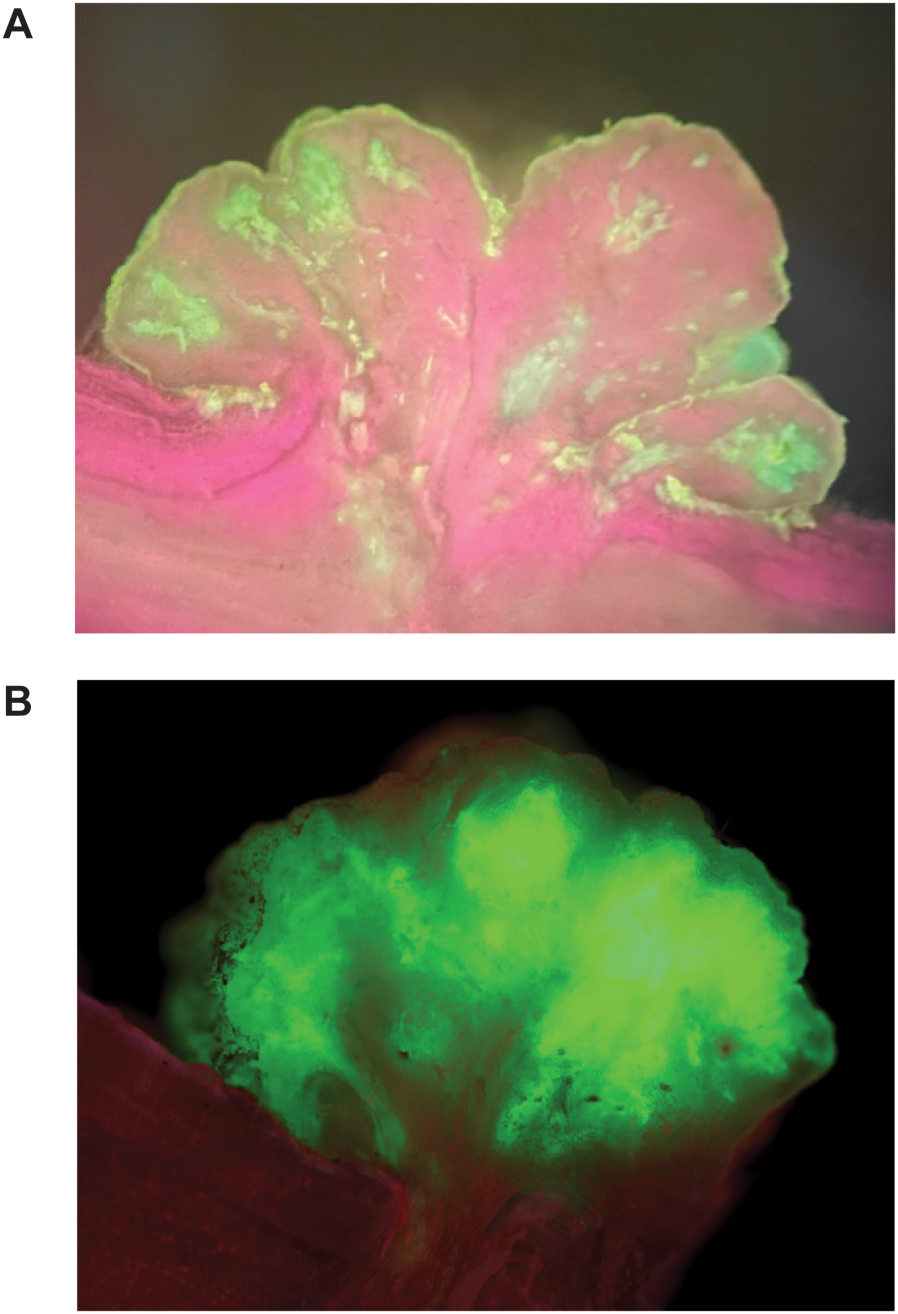
Heterologous protein expression in symbiont tissue. Similar to RUBY, protein expression patterns can appear **(A)** patchy in small niches of cell division or **(B)** more homogeneously dispersed throughout the tissue.

### Small molecules can be produced by and accumulate in symbionts

Small molecules can also be recombinantly produced and collected in symbionts. When we formed symbionts on citrus trees expressing the three-gene pathway for the red pigment reporter RUBY (betalain) (He et al., 2020), we observed visible pigment accumulation in the symbiont (**Figure 5B**). When we formed pSym-derived symbionts on the potato cultivar NY129 ‘Red Maria’, which naturally accumulates high levels of anthocyanins within tuber skins, we sometimes observed pronounced anthocyanin accumulation in the symbiont (**Supplementary Figure S3A**). This high-level of accumulation was less often observed in pSym-derived symbionts on the cultivar Desiree (**Supplementary Figure S3B**), demonstrating that genomically-encoded pigments can accumulate in symbionts from altered host-cell expression or metabolic profiles (**Table 1**).

### Symbionts can be used to produce proteins *in vitro*

**Figure 7:**
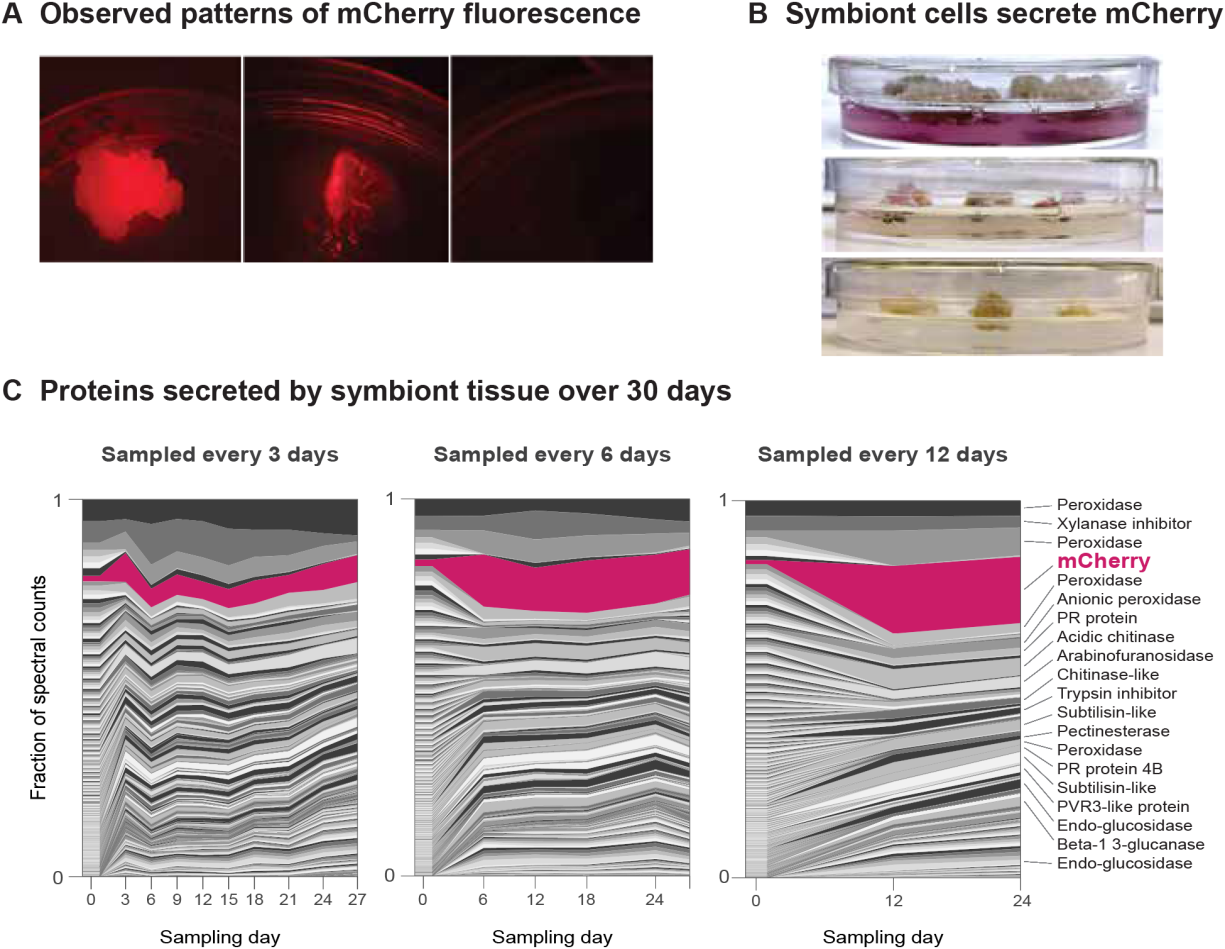
Protein production from symbiont cells in tissue culture. **(A)** Fluorescence of mCherry in friable tomato pSym-SP-FT-mCherry symbiont tissue (left), in the agar where the symbiont tissue was growering (center) vs fresh media (right). **(B)** Top plate shows tissue culture of friable tomato pSym-SP-FT-mCherry symbiont tissue. Middle plate shows compact tomato pSym-SP-FT-mCherry symbiont tissue and bottom plate shows Friable tomato pSym tissue expressing an antimicrobial peptide (no mCherry fusion). **(C)** Fraction of total spectral counts for 1733 tomato proteins and FT-mCherry observed in total proteomics of tomato pSym-SP-FT-mCherry SFI and the liquid removed from solid media used to culture the symbiont cells. Three sets of triplicate cultures were sampled every 3, 6, or 12 days to monitor protein accumulation for 30 days, with cells harvested for proteomics at the end of the experiment. Short descriptions of the top 20 most abundant (by total spectral counts) proteins in the 12 day sampled supernatants are listed. A full list of proteins detected and spectral counts for each sample is given in the supplemental data set file.

The pSym plasmid can be used to transform callus tissue (**Figure 2B**) or established symbionts can be removed from the plant and the tissue maintained in tissue culture (**Figure 2B**). Serial passage of symbiont tissue from tomato pSym-GFP and pSym-SP-FT-mCherry symbionts on kanamycin plates visibly improved homogeneity and intensity of transgene expression (**Supplementary Figure S4**). We observed that symbiont tissue tended to consist of a mixture of two distinct morphologies commonly observed in plant cell tissue culture; compact and friable (Ikeuchi et al., 2013; Ashrafadeh et al., 2015). We found that friable symbiont tissue could be generated from compact symbiont tissue, but not vice-versa. We unexpectedly observed that a significant amount of mCherry protein accumulated in the culture media of friable cells of tomato pSym-SP-FT-mCherry symbiont forming inoculum (**Figure 7A**). Pigment accumulation in the media was visibly higher for cultures of friable cells (**Figure 7B** top) than compact cells incubated for the same period (**Figure 7B** middle) and no such pigmentation was observed in control cultures of tomato cells transformed with a pSym plasmid expressing a small antimicrobial peptide (**Figure 7B** bottom). Media with accumulated pigment fluoresces in the expected color range (**Figure 7A**), confirming that at least some of the mCherry protein is both intact and properly folded.

A mass spectrometry analysis of both the symbiont tissue and protein extracted from the solid culture medium showed that tryptic peptides from mCherry were highly abundant in the cells, relatively enriched in the culture medium and increasingly so when allowed to accumulate over three, six, or twelve days (**Figure 7C**) with mCherry comprising 7%, 12%, and 17% percent of total spectral counts, respectively. These results confirm secretion into the culture medium and demonstrate the potential for use of symbiont tissue as an *in-vitro* protein expression system. While optimization on this aspect is still required, symbiont tissue may be scaled up for larger-volume protein production.

The proteomics data had notable relative abundance of plant proteins with expected function in both cell wall modification and pathogen response, particularly the proteins relatively enriched in the culture medium (**Figure 7C**). These proteins are particularly dominated by peroxidases, collectively comprising over 15% of total spectral counts in the 12-day supernatant samples, with proteases, chitinases, and cell wall polysaccharide cleaving enzymes also prominent. These proteins are consistent with previous descriptions of callus-tissue proteomes (Yin et al., 2007; Tan et al., 2013) showing the biochemical profiles of symbiont tissues are quite similar to transformed callus growing in tissue culture.

### Symbiont burden on plant growth and fruit production is low

To test if symbionts had any detrimental effects on plant growth and development, we inoculated 1-month old tomato plants with four symbionts each and monitored plant growth in controlled environmental chambers. There was no statistically significant change in plant height, branch length, number of flowers or number of tomato fruit produced (**Supplementary Figure S5**), though plants with symbionts averaged a slightly higher number of fruit produced (*P*-value = 0.11). We tested for possible growth burden of wild-type galls in the field. We inoculated young potato plants (cultivars Atlantic and Woodland) with a wild *Agrobacterium* isolate (strain 1D159) (Huo et al., 2019), disarmed EHA105, or a mock inoculation with no bacterium at four sites on the main stem. At harvest time there was no significant decrease observed for total weight of tubers per plant or for total number of tubers per plant (**Supplementary Figure S6**). If any effect was observed, there may have been a small increase in the total weight of tubers for plants with galls and/or plants inoculated with disarmed agrobacterium, but the difference was not statistically significant. These results showed that at least under some circumstances, gall growth is not sufficiently burdensome on crops to compromise productivity.

### Vascular connections to the host are functional, but product movement is restricted

**Figure 8:**
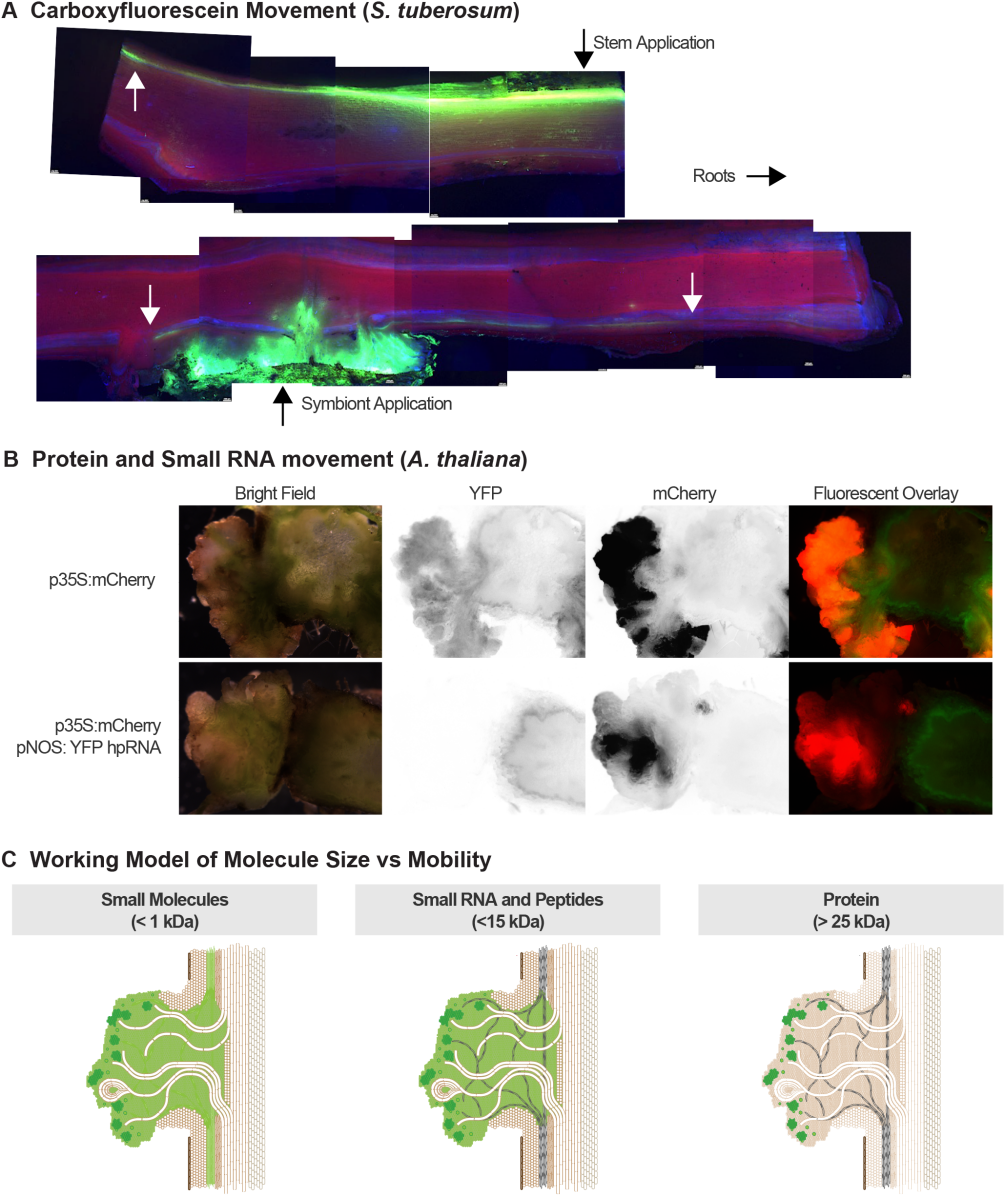
Movement of small molecules, small RNAs and proteins in symbionts. **(A)** Fluorescent microscopy of longitudinal section of potato stem after external carboxyfluorescein diacetate application to a wounded stem (top) or an exposed surface of a cut symbiont (bottom). **(B)** Fluorescent microscopy of cross-sections of transgenic *Arabidopsis* expressing YFP from the PIN4 promoter bearing symbionts expressing mCherry from the *A tumefaciens* NOS promoter with or without expression of a YFP-silencing hairpin RNA from the CaMV35S promoter. **(C)** Working model of size-limitations on macromolecule movement within symbionts and export to the host plant. Small molecules move freely throughout the symbiont and into the vasculature of the host plant. Small RNAs and small peptides move freely throughout the symbiont but may be limited in either entry into the vasculature within the symbiont or into the stem vasculature. Larger proteins remain concentrated in clusters of highly expressing cells within the symbiont and may be mobile throughout the symbiont and into the host vasculature, but at greatly reduced concentration.

While it is long established that small molecules and viral particles can enter wild type galls through their vascular connection to the plant (Pradel et al., 1999), we conducted some preliminary studies of the functionality of the well-developed vascular connections we observed in symbionts formed on various plant species. When the phloem-labeling dye CFDA was introduced to the wounded stem of a potato plant and then longitudinal cross sections of stem were observed with a fluorescence microscope, we observed characteristic fluorescence of the contiguous phloem on that side of the plant (**Figure 8A**). When CFDA was applied to the cut surface of symbionts on potato, the entire symbiont mass was fluorescent and in two out of four plants examined, characteristic staining of the host phloem was observed above and below the symbiont growth site, although for a shorter distance and at lower intensity than observed for direct stem application (**Figure 8A**). These observations are consistent with functional vascular connections to the host plant, albeit with a slower rate of transport out of the symbiont than within the primary vasculature of the stem. The inconsistency of vascular labeling among the different symbionts observed may be a function of variation in the capacity of the vasculature, or in the strength and consistency of vascular flow into the symbiont which has mainly been observed as a sink tissue for phloem and xylem flow (Schurr et al., 1996).

Having noted heterogeneous regions of fluorescent protein expression, suggesting at least some restriction of protein movement within the symbiont, we hypothesized that small RNAs might be more mobile. To test this, we constructed a symbiont vector expressing a YFP-silencing hairpin construct under CaMV35S promoter and cytoplasmic mCherry under the NOS promoter and used a strain bearing this vector to inoculate an *A. thaliana* line that expresses an immobile (cell-autonomous) 4-copy YFP protein from the PIN4 promoter. Symbionts not expressing the YFP hpRNA construct express YFP throughout the symbiont and adjacent stem (**Figure 8B**), whereas symbionts expressing the hpRNA had very low YFP signal throughout the symbiont and not only in areas of high mCherry expression, demonstrating movement of the silencing signals throughout symbiont tissue. This pattern was consistent across four independent experiments with a total of 28 plants (*P* < 0.0001, see **Supplementary Figure S7**). There was still significant YFP expression in the stem in all cross-sections evaluated; however, there was a slightly lower average intensity adjacent to the hpRNA expressing symbionts (*P* = 0.0003). Taken together these results suggest a size-dependent hierarchy of movement between the transgene expressing symbiont tissue, non-expressing symbiont tissue and the connecting vasculature (**Figure 8C**, **Table 1**).

## Discussion

The symbiont concept is a new approach to plant bioengineering with future potential applications in biomolecule production on whole plants and *in vitro.* Our work to use the *A. tumefaciens* PGR cassette to metabolically reprogram certain plant cell types for autonomous cell division and transgene expression builds on decades of observations that underline the complexity of interaction amongst differing combinations of *A. tumefaciens* genomes, Ti plasmid composition, and plant genomes mediating successful infection and T-DNA integration. This formed a semi-organized plant cell structure connected and growing externally to the host plant, providing practical advantages to modifying phenotypes of established trees as compared to traditional, whole-plant transgenic modification. Accordingly, future research should be focused on identifying the optimum set of genes regulating symbiont cell proliferation for each plant species. The general approach may be used to modify host plant phenotypes or to produce biomolecules. For *on plant* symbiont structures to modify host plant phenotype, we hypothesize that biomolecules must be produced by symbiont tissues on the plant and delivered to the host plant, either directly via an export mechanism or indirectly via systemic transmission of signaling molecules generated in the symbiont. Additionally, modification of host phenotypes may be due to modification of the local environment near the symbiont or in sites distal to the symbiont, such as roots and shoots. In this concept, symbionts may be used to induce plant defenses, deliver antimicrobial peptides or modify other phenotypic traits of interest. Transient reprogramming of host plant phenotypes has already been achieved using viral vectors (Torti et al., 2021). Symbiont cells can also be used to produce and harvest biomolecules *in vitro* or on plants. Molecules produced by symbionts can be further purified and used for downstream applications. These downstream applications may be in agriculture, such as trunk injection, or in animal health, such as in the production of biologics (Rivnay et al., 2024).

Current symbiont-forming methods rely on the use of *A. tumefaciens* to deliver the pSym plasmid into plant cells and initiate plant cell transformation. This raises the question as to whether the technology will be regulated as a modified microbial biopesticide, a transgenic plant, or some new framework that does not yet exist. Studies to define the systemic movement of *A. tumefaciens* in symbiont-inoculated plants will generate data supportive of that distinction. Similarly, the survival of the *A. tumefaciens* in symbiont tissue could be important for certain applications. When *Agrobacterium* is used to deliver symbiont-forming plasmids to plants, there will undoubtedly be a period of time where the disarmed *A. tumefaciens* will survive in symbiont tissues despite the lack of opine synthesis. The length of time of agrobacterium survival most likely will depend on interactions between the bacterial strain and the host plant. The use of available auxotrophic strains of *A. tumefaciens* (Collens et al., 2004; Prías-Blanco et al., 2022) is expected to greatly reduce the survival of *A. tumefaciens* in symbiont tissue. Alternatively, encoding secreted antimicrobial peptides into the symbiont plasmid that specifically target *A. tumefaciens* could help to more rapidly clear the live bacteria from symbiont tissues. Likewise, use of other plasmid delivery strategies such as particle bombardment or nanoparticle carriers (Dunbar et al., 2022) may eliminate the need to use *A. tumefaciens* completely.

Peptide or biomolecule export from the symbiont and bioproduct activity are also major frontiers of research. Heterogenous fluorescence within *in-vitro* symbiont cell clusters resulted in significant mCherry protein export and accumulation in the extracellular medium over time. But on plants, protein export from the symbiont to the host vascular and from transgenic cells to non-transgenic cells within a given symbiont is more tightly controlled. Our observations align with other studies examining transient expression of reporter genes in the wood of perennials, where heterogeneity was also observed in stem tissue, including cambial cells (Spokevicius et al., 2005). Our observations of heterogeneous protein expression within the symbiont are suggestive of at least some restriction of protein movement out of the transgene-expressing cells. Small RNAs on the other hand, were sufficiently mobile as to consistently silence locally expressed YFP outside of the cell niches expressing the hairpin constructs from which they were derived. Thus, we see evidence of a hierarchy of movement restriction by molecule size within the symbiont and in the vascular connections between symbiont and host plant. Further investigation of these movement restrictions will be an important area of research to enable effective use of symbiont technology. We note that symbionts offer relative speed and ease to generate tissues with diverse genotypes that can over-express or silence native genes involved in gall development, vascular maturation, or plasmodesmata development and function. These abilities position symbionts as an excellent tool to explore these fundamental elements of plant physiology and development outside of soft leafy tissues.

Understanding symbiont longevity will guide how the technology may be used for plant pest and disease management. When used in agriculture, and especially when treating diseases perennial fruit trees, important questions still remain. Will growers have to apply a new symbiont every year to a tree? Will a single symbiont be sufficient for the life of the tree or even a single growing season? The use of inducible promoters may provide some level of control of transgene expression, which could be critical to manage residue concerns. How might the technology be applied to row crops or to monocots?

The PGR cassette delivered by pSym has no opine synthesis genes and therefore should theoretically pose minimal metabolic burden on the host plant. The resources required for symbiont growth might still reduce plant growth or productivity, especially under stressful conditions, because of the formation of a strong sink at the site of inoculation and symbiont formation. Thus, for symbionts to have any practical utility in agriculture, there must be treatment regimens under which any such burden is small compared to the benefit conferred by a symbiont-delivered therapeutic. In multiple experiments in the greenhouse and the field, we have observed this condition to be met, even with symbionts formed by wild *Agrobacterium* isolates, with no measurable decrease in plant growth or fruit production. However, we have observed cases in which individual plants have died after inoculation with multiple symbionts. Our observations, coupled with wildly divergent quantification of wild agrobacterium’s impact on various plant species (Garrett, 1987; Schroth, 1988) reinforce the expectation that as with any crop treatment, the timing, method, and intensity of symbiont inoculation will need to balance the specific benefits of a delivered therapeutic molecule and the needs of the target crop to optimize horticultural outcomes.

Our work showed that symbiont cells can be used to produce protein *in vitro*, which paves the way for the technology to also produce smaller, antimicrobial peptides and other small biomolecules using this system. For controlling citrus greening disease in Florida, citrus growers have shown that treating individual trees using injectable antibiotics is feasible (Albrecht et al., 2025). Direct injection of therapies into trees couples naturally to plant-produced therapies in the symbiont *in vitro* system. Our observations on the production of anthocyanins and RUBY in symbionts demonstrate the potential for the exploitation of symbionts on plants as collection points for the end products of natural and recombinant metabolic pathways for high value small molecules, leveraging the solar energy harvesting and carbon-capture capacities of the entire host plant to deliver concentrated product for more efficient harvest and extraction.

We hypothesize that the symbiont approach may enable a future ‘Smart Plant’ platform, enabling engineering of plant response to threats in real time by producing a suite of therapies specific to the threat encountered that may overcome the plant’s innate response or act synergistically with it. Development of symbionts to solve citrus greening and other diseases caused by plant vascular pathogens may provide long-term, sustainable solutions, reduce reliance on spray application of pesticides, provide a platform for the delivery of peptide and RNA based therapeutics and be an effective alternative to whole plant transgenic crops. Research incorporating knowledge from studies on long-distance signaling in plants (Cai et al., 2023; Kondhare et al., 2025) will be critical to further advance the technology as a crop protection tool. Application of the strategy on plants could be developed to introduce genome editing reagents into plant species for which tissue-culture regeneration of whole plants is not yet possible. While these latter ideas are theoretical at this time, we hypothesize this new paradigm in plant biotechnology has potential to synergistically accelerate crop development in combination with traditional breeding and plant genome engineering.

## Data Availability

The mass spectrometry proteomics data have been deposited to the ProteomeXchange Consortium via the PRIDE partner repository with the dataset identifier PXD071454 and 10.6019/PXD071454. All other data are available in the **Supplementary Material**.

## Supplementary material

Supplementary Figure 1 Symbiont tissue growth on citrus is enhanced by wrapping the inoculation site. **(A)** An early method of cutting the bark prior to inoculation resulted in the formation of small symbionts that took ~6 months to grow (left). In contrast, wrapping symbiont inoculation sites with an initial layer of parafilm and black insulation tape resulted in faster and more robust symbiont tissue growth (right). Symbiont structures shown here are 1 month post-inoculation using the wrapping method expressing the RUBY reporter. **(B)** Wrapping symbiont inoculation sites with an initial layer of parafilm and black insulation tape results in larger symbiont growth in a much shorter period of time.

Supplementary Figure 2 Symbiont growth in plants using the transplantation method is feasible on tomato, where *Agrobacterium tumefaciens* is first cleared by tissue passage on antibiotic selection. Shown is symbiont formation after transplantation from tissue culture on tomato, where *Agrobacterium tumefaciens* was cleared by tissue passage on antibiotic selection. Success in transplantation most often occurred when the end of the stem was split in half as opposed to putting a plug of tissue in the side of the stem, as we did for inoculation with *A. tumefaciens.* Ruler scale is in millimeters.

Supplementary Figure 3 Symbionts can be used to make genomically-encoded plant natural products. **(A)** The pSym-derived symbionts on the potato cultivar NY129 ‘Red Maria’, which naturally accumulates high levels of anthocyanins within tuber skins, often showed pronounced anthocyanin accumulation. **(B)** This high-level of accumulation was less often observed in pSym-derived symbionts on the cultivar Desiree.

Supplementary Figure 4 *In vitro*-grown symbiont tissue can be selected for homogeneity in transgene expression in callus tissue. Shown is symbiont callus tissue transformed with a pSym plasmid expressing an mCherry fusion protein.

Supplementary Figure 5 Growth and fruit production of tomato plants is not negatively impacted by symbionts. Tomato Lanai bearing four symbionts per plant grown in greenhouse chambers in three independent experiments. 5 to 8 plants per experiment, 19 total plants per treatment, 38 plants total. **(A)** Plant heights by week for experiments 1 and 3 (N = 13). (Height and branch length measurements were not taken in experiment 2) **(B)** Average branch lengths at week 4 for experiments 1 and 3 (N = 13). T-test *P-*value = 0.38. **(C)** Number of flowers for all experiments (N = 19). T-test on week 5 counts, *P-*value = 0.81. **(D)** Number of fruit for all experiments (N = 19). T-test on week 5 counts, *P-*value = 0.11. See supplemental data set for full data.

Supplementary Figure 6 Tuber production for potato field trail for Woodland and Atlantic cultivars inoculated with wild type *Agrobacterium fabrum* 1D159, disarmed *A. tumefaciens* EHA105 (no symbiont structure formation), or injected with the induction buffer only (Mock). Number of plants harvested: Atlantic EHA105 = 49, Atlantic Mock = 58, Atlantic Wild Type = 53, Woodland EHA105 = 31, Woodland Mock = 45, Woodland Wild Type = 38. **(A)** Total tuber weight (ANOVA *P-*values: Treatment = 0.01, Cultivar = 0.80, Treatment: Cultivar = 0.08) **(B)** Number of tubers per plant. (ANOVA *P-*values: Treatment = 0.42, Cultivar = 0.002, Treatment: Cultivar = 0.20). Data were collected from a single growing season and can be found in the supplemental dataset provided.

Supplementary Figure 7 Initiation of RNA silencing in symbionts allows modification of symbiont gene expression. Plant averages for YFP intensity (as measured by fluorescence microscopy) in the symbiont and adjacent stem tissue are plotted in **(A, B)** respectively. For each plant, 2–10 cross sections of the symbiont and host stem were analyzed. Data from four independent experiments, with 2–5 plants per treatment per experiment (28 plants total). See supplemental data set for full data.
