# Supplementary figures and images for: A new use of Agrobacterium plant growth regulator genes for plant bioengineering
Source: Front Plant Sci. 2026 Mar 16;17:1754357. doi: 10.3389/fpls.2026.1754357 (PMC13033780; doi:10.3389/fpls.2026.1754357)

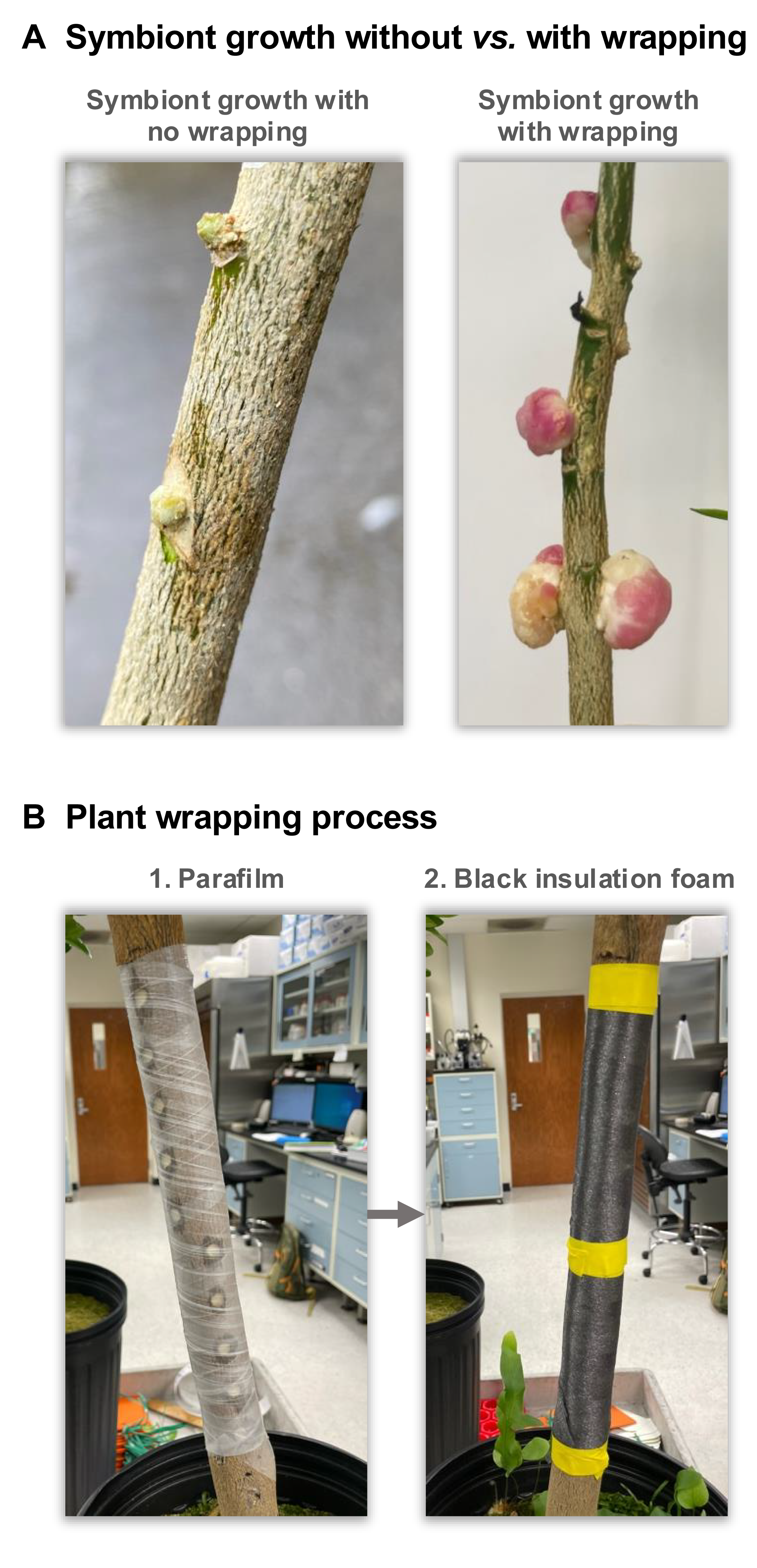

Supplement: Supplementary Figure 1 — Symbiont tissue growth on citrus is enhanced by wrapping the inoculation site. (A) An early method of cutting the bark prior to inoculation resulted in the formation of small symbionts that took ~6 months to grow (left). In contrast, wrapping symbiont inoculation sites with an initial layer of parafilm and black insulation tape resulted in faster and more robust symbiont tissue growth (right). Symbiont structures shown here are 1 month post-inoculation using the wrapping method expressing the RUBY reporter. (B) Wrapping symbiont inoculation sites with an initial layer of parafilm and black insulation tape results in larger symbiont growth in a much shorter period of time. [file Image1.tif]

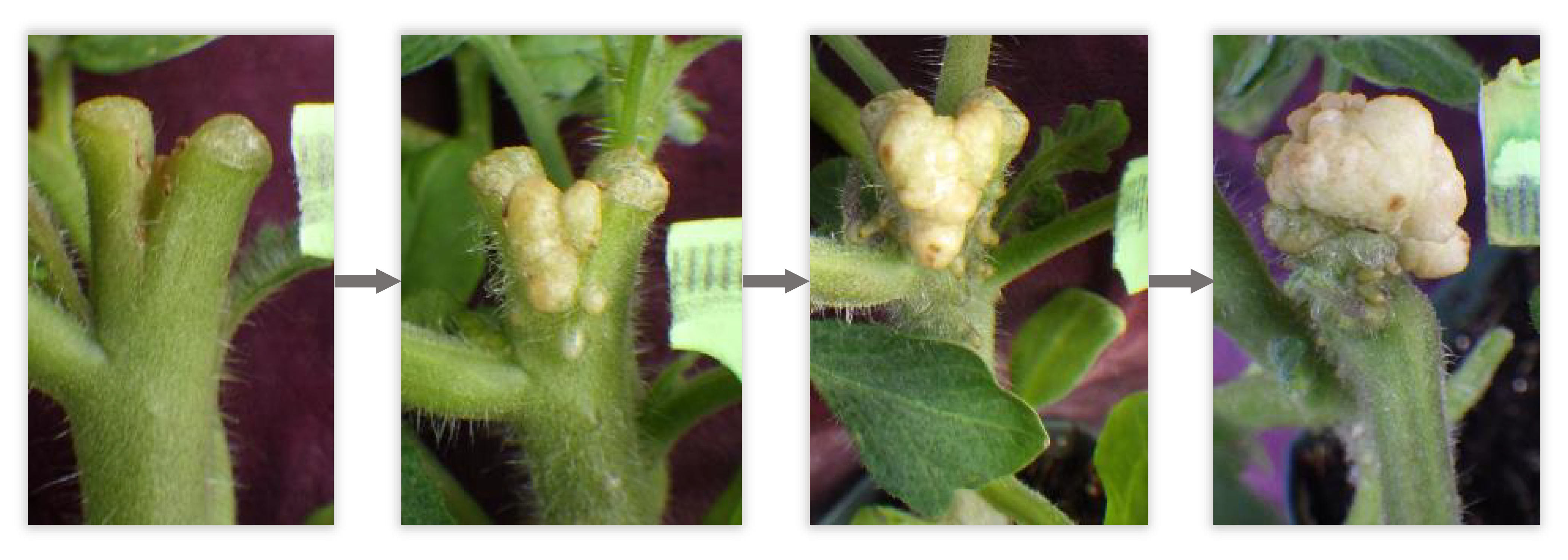

Supplement: Supplementary Figure 2 — Symbiont growth in plants using the transplantation method is feasible on tomato, where Agrobacterium tumefaciens is first cleared by tissue passage on antibiotic selection. Shown is symbiont formation after transplantation from tissue culture on tomato, where Agrobacterium tumefaciens was cleared by tissue passage on antibiotic selection. Success in transplantation most often occurred when the end of the stem was split in half as opposed to putting a plug of tissue in the side of the stem, as we did for inoculation with A. tumefaciens. Ruler scale is in millimeters. [file Image2.tif]

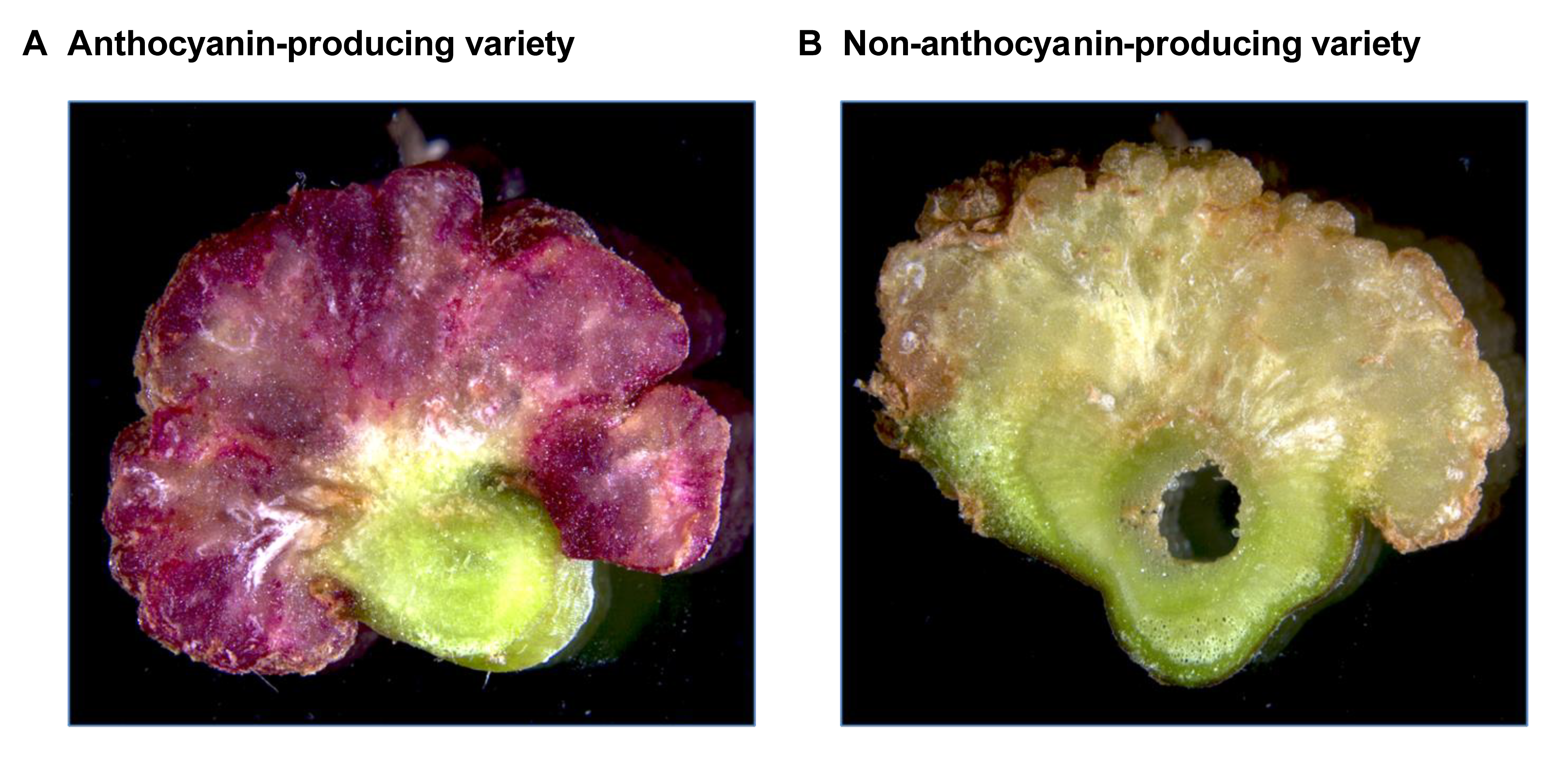

Supplement: Supplementary Figure 3 — Symbionts can be used to make genomically-encoded plant natural products. (A) The pSym-derived symbionts on the potato cultivar NY129 ‘Red Maria’, which naturally accumulates high levels of anthocyanins within tuber skins, often showed pronounced anthocyanin accumulation. (B) This high-level of accumulation was less often observed in pSym-derived symbionts on the cultivar Desiree. [file Image3.tif]

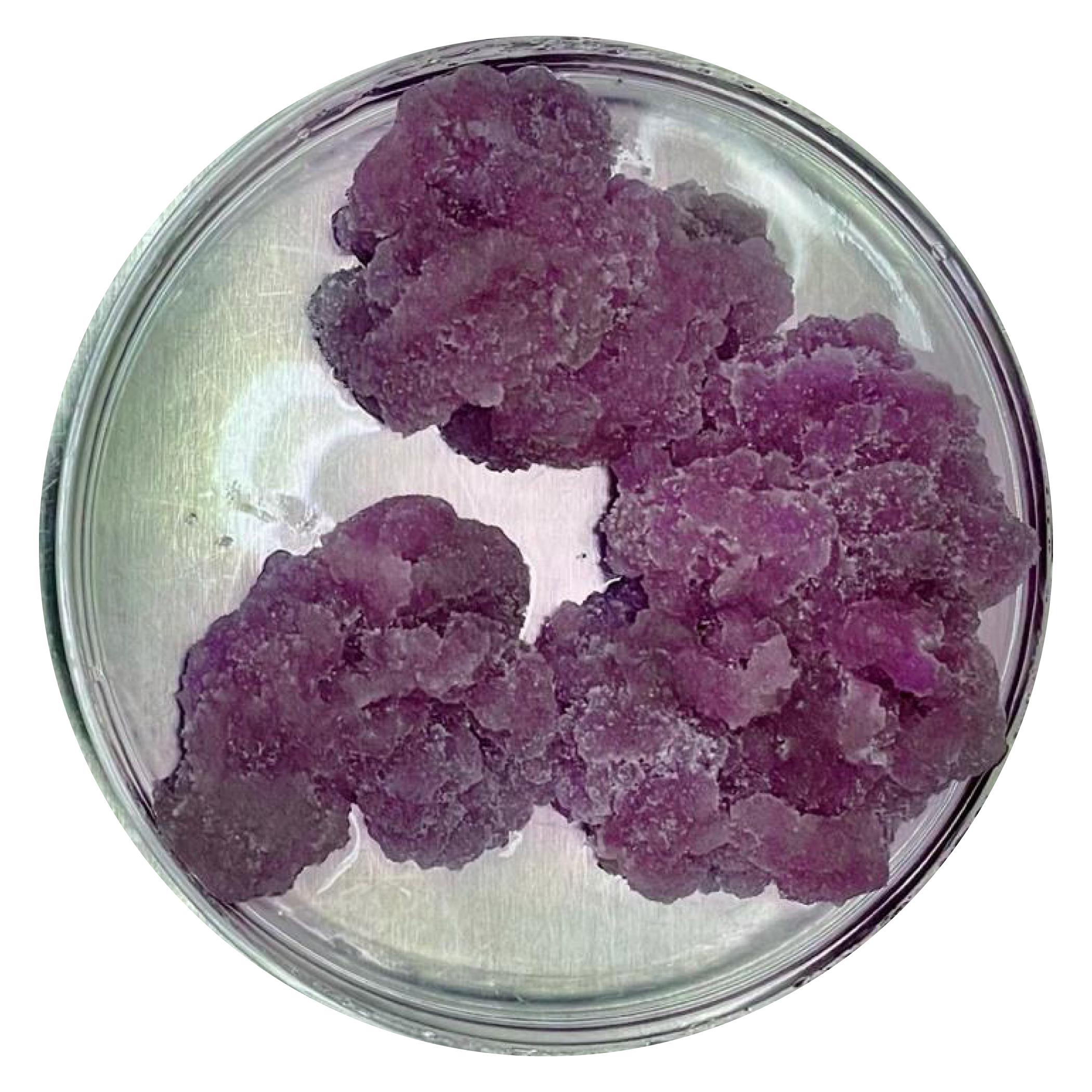

Supplement: Supplementary Figure 4 — In vitro-grown symbiont tissue can be selected for homogeneity in transgene expression in callus tissue. Shown is symbiont callus tissue transformed with a pSym plasmid expressing an mCherry fusion protein. [file Image4.tif]

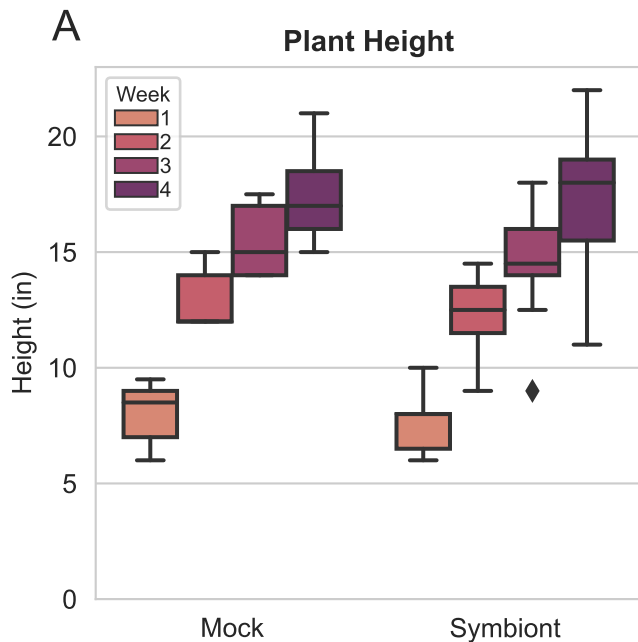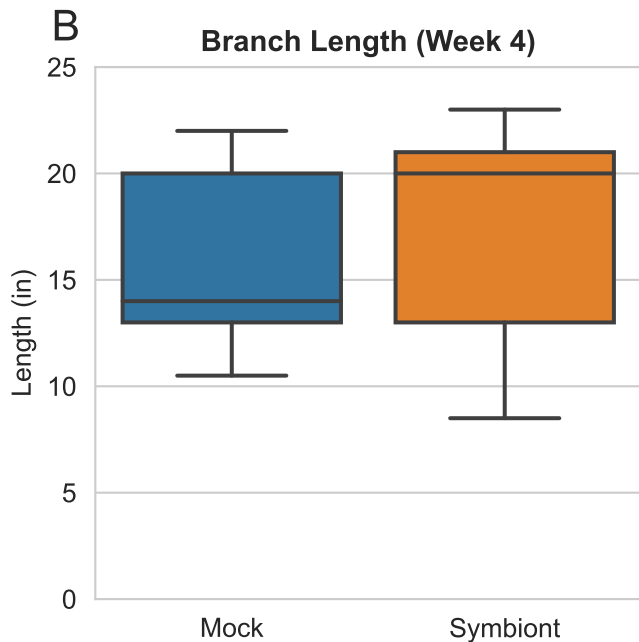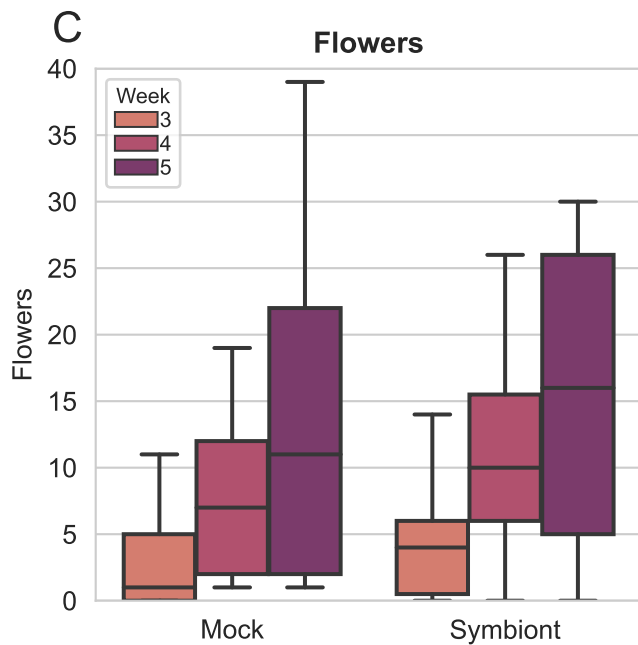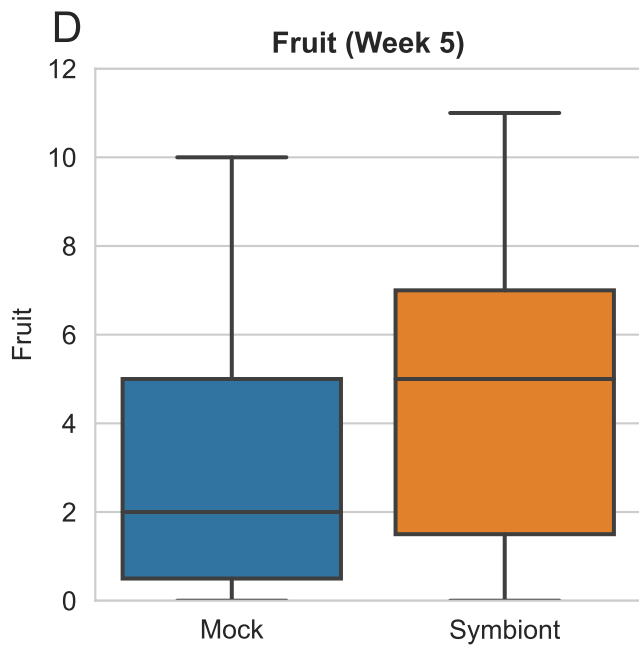

Supplement: Supplementary Figure 5 — Growth and fruit production of tomato plants is not negatively impacted by symbionts. Tomato Lanai bearing four symbionts per plant grown in greenhouse chambers in three independent experiments. 5 to 8 plants per experiment, 19 total plants per treatment, 38 plants total. (A) Plant heights by week for experiments 1 and 3 (N = 13). (Height and branch length measurements were not taken in experiment 2) (B) Average branch lengths at week 4 for experiments 1 and 3 (N = 13). T-test P-value = 0.38. (C) Number of flowers for all experiments (N = 19). T-test on week 5 counts, P-value = 0.81. (D) Number of fruit for all experiments (N = 19). T-test on week 5 counts, P-value = 0.11. See supplemental data set for full data. [file Image5.pdf]

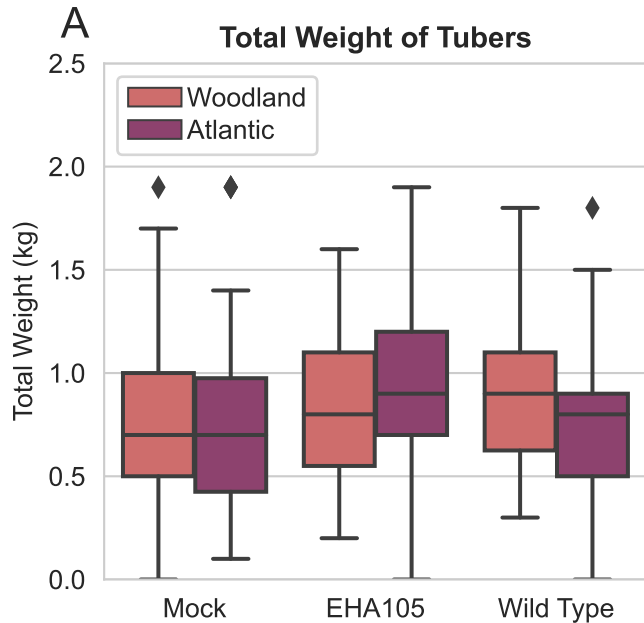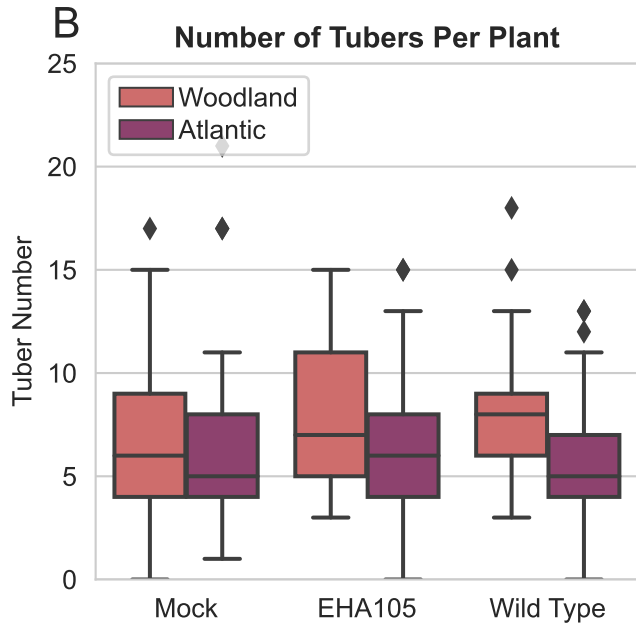

Supplement: Supplementary Figure 6 — Tuber production for potato field trail for Woodland and Atlantic cultivars inoculated with wild type Agrobacterium fabrum 1D159, disarmed A. tumefaciens EHA105 (no symbiont structure formation), or injected with the induction buffer only (Mock). Number of plants harvested: Atlantic EHA105 = 49, Atlantic Mock = 58, Atlantic Wild Type = 53, Woodland EHA105 = 31, Woodland Mock = 45, Woodland Wild Type = 38. (A) Total tuber weight (ANOVA P-values: Treatment = 0.01, Cultivar = 0.80, Treatment: Cultivar = 0.08) (B) Number of tubers per plant. (ANOVA P-values: Treatment = 0.42, Cultivar = 0.002, Treatment: Cultivar = 0.20). Data were collected from a single growing season and can be found in the supplemental dataset provided. [file Image6.pdf]

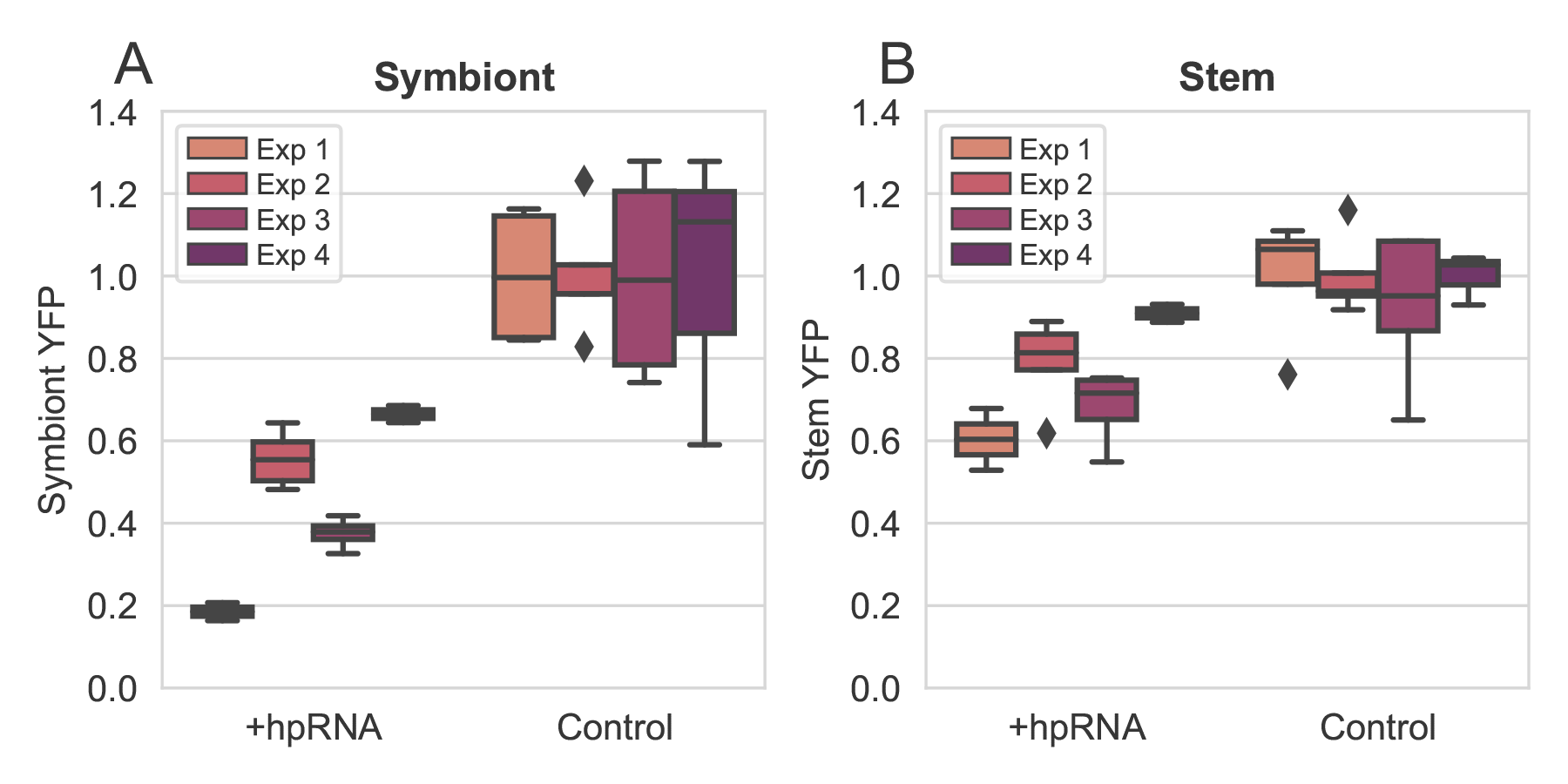

Supplement: Supplementary file 7 [file Image7.tif]
